# Development of a method for the synthesis of 2,4,5-trisubstituted oxazoles composed of carboxylic acid, amino acid, and boronic acid

**DOI:** 10.3762/bjoc.13.146

**Published:** 2017-07-27

**Authors:** Kohei Yamada, Naoto Kamimura, Munetaka Kunishima

**Affiliations:** 1 Faculty of Pharmaceutical Sciences, Institute of Medical, Pharmaceutical, and Health Sciences, Kanazawa University, Kakuma-machi, Kanazawa 920-1192, Japan

**Keywords:** one-pot oxazole synthesis, Suzuki–Miyaura coupling, triazine, 5-(triazinyloxy)oxazole, trisubstituted oxazole

## Abstract

A novel method for the synthesis of trisubstituted oxazoles via a one-pot oxazole synthesis/Suzuki–Miyaura coupling sequence has been developed. One-pot formation of 5-(triazinyloxy)oxazoles using carboxylic acids, amino acids and a dehydrative condensing reagent, DMT-MM, followed by Ni-catalyzed Suzuki–Miyaura coupling with boronic acids provided the corresponding 2,4,5-trisubstituted oxazoles in good yields.

## Introduction

**Scheme 1:**
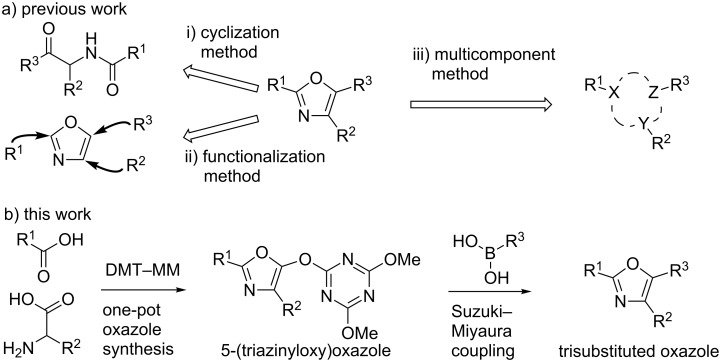
Our strategy for the concise synthesis of 2,4,5-trisubstituted oxazoles.

Oxazoles are found in numerous natural products and are used as a broad range of artificial compounds [1–2]. In particular, 2,4,5-trisubstituted oxazoles attract attention as pharmacologically potent scaffolds because structural diversity can be efficiently generated by the introduction of a variety of substituents. Accordingly, numerous synthetic methods have been developed and can be roughly classified into three synthetic strategies (Scheme 1a).

i) The cyclization method: many methods, such as the Robinson–Gabriel oxazole synthesis using α-acylaminoketone [3–4], the Davidson oxazole synthesis with α-acyloxyketone [5], and modifications of these [6–7], have been developed. Moreover, cycloaddition of two starting materials, such as α-haloketones and primary amides [8], alkynes and nitriles [9], amines and α,β-unsaturated carbonyl compounds [10], etc. [11] have been reported. However, these reactions are often conducted under harsh reaction conditions and multistep syntheses of the starting materials are needed. ii) The functionalization method: various regioselective metalations and subsequent functionalizations of the oxazole core skeleton using Cu [12], Pd [13], Mg [14], Zn [14], etc. [15] have been developed. This linear synthetic approach inevitably requires multistep processes and often needs prehalogenation [16]. iii) The multicomponent method: only two strategies have been reported to the best of our knowledge. One is a combination of the Ugi reaction, which uses 2,4-dimethoxybenzylamine, arylglyoxal, carboxylic acid, and isonitrile as components, and a subsequent Robinson–Gabriel reaction [17]. The other is an Au-catalyzed tandem oxazole synthesis using a primary amide, aldehyde, and alkyne [18]. These methods are reasonable for the synthesis of diverse libraries of trisubstituted oxazoles because the combination of three starting materials that are corresponding to the substituents can be readily altered. However, these reactions require strongly acidic conditions or high temperatures. Therefore, a mild method for the synthesis of diverse trisubstituted oxazoles using three commercially available compounds with a wide variety of structures is still desired.

Previously, we reported a one-pot synthesis of oxazolone from carboxylic acids and amino acids using a dehydrative condensing reagent, 4-(4,6-dimethoxy-1,3,5-triazin-2-yl)-4-methylmorpholinium chloride (DMT-MM [19–22])[23]. Formation of 5-(triazinyloxy)oxazole is also reported to occur when an excess of DMT-MM was used. Recently, Jin and co-workers reported that Ni-catalyzed Suzuki–Miyaura coupling between triazinyloxybenzene and arylboronic acids affords the corresponding biaryl compounds [24–33]. In this context, we envisioned application of this Suzuki–Miyaura coupling to a 5-(triazinyloxy)oxazole would provide trisubstituted oxazoles (Scheme 1b). Since many kinds of carboxylic acids, amino acids and boronic acids, which are corresponding to 2-, 4-, and 5-substituents of the oxazole, respectively, are commercially available, this method is suitable for the synthesis of a diverse variety of trisubstituted oxazoles. Herein, we described an efficient method for the synthesis of trisubstituted oxazoles through a one-pot oxazole synthesis and subsequent Suzuki–Miyaura coupling.

## Results and Discussion

The study was initiated with the preparation of the key intermediate, 5-(triazinyloxy)oxazole **3**, from carboxylic acid **1** and amino acid **2** under conditions improved from [23] (Table 1). A one-pot sequence involving formation of an activated ester from benzoic acid (**1a**) with DMT-MM, *N*-benzoylation of alanine (**2a**), cyclodehydration, and introduction of the triazinyl group was conducted in 1,4-dioxane/H_2_O to give the desired 5-(triazinyloxy)oxazole **3aa** in 78% yield (Table 1, entry 1). A series of carboxylic acids were subjected to the reaction conditions. Aromatic carboxylic acids with both electron-withdrawing and electron-donating groups gave good yields (Table 1, entries 2 and 3). In the case of aliphatic carboxylic acids, 3-phenylpropionic acid (**1d**) gave a slightly decreased amount of **3da** in 54% yield (Table 1, entry 4), whereas the more sterically demanding **1e** gave the desired product **3ea** in a good yield (Table 1, entry 5). The reaction was carried out with different amino acids, resulting in a varied substitution pattern at the 4-position of the oxazole. The one-pot oxazole synthesis with phenylalanine (**2b**), valine (**2c**), leucine (**2d**), methionine (**2e**), and phenylglycine (**2f**) proceeded to give the corresponding intermediates in good yields (Table 1, entries 6–10). Despite the existence of highly polar starting materials and relatively more lipophilic activated esters and oxazolone intermediates during the course of the reaction, various 5-(triazinyloxy)oxazoles were uneventfully synthesized under these improved conditions.

**Table 1:** One-pot synthesis of 5-(triazinyloxy)oxazole under improved conditions.

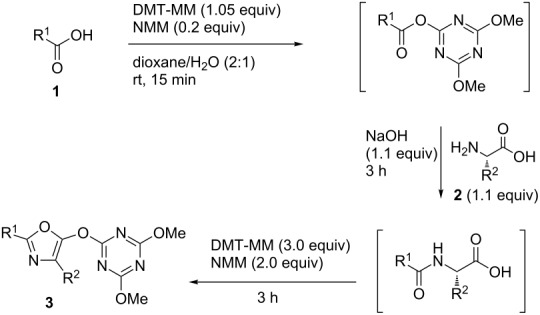			

entry	carboxylic acid **1**	amino acid **2**	yield of **3** (%)^a^

1	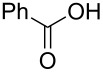 **1a**	alanine (**2a**)	**3aa**, 78
2	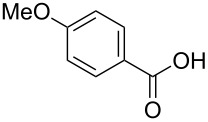 **1b**	**2a**	**3ba**, 69
3	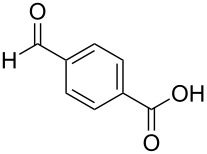 **1c**	**2a**	**3ca**, 60
4	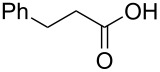 **1d**	**2a**	**3da**, 54
5	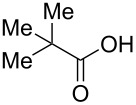 **1e**	**2a**	**3ea**, 71
6	**1a**	phenylalanine (**2b**)	**3ab**, 78
7	**1a**	valine (**2c**)	**3ac**, 83
8	**1a**	leucine (**2d**)	**3ad**, 70
9	**1a**	methionine (**2e**)	**3ae**, 70
10	**1a**	phenylglycine (**2f**)	**3af**, 78

^a^Isolated yield.

According to the procedure previously reported [24], we examined Ni-catalyzed Suzuki–Miyaura coupling between 5-(triazinyloxy)oxazole **3aa** and phenylboronic acid (**4a**, Table 2). As expected, the desired trisubstituted oxazole **5aaa** was obtained in 21% yield (Table 2, entry 1). The use of different bidentate (dppp) or monodentate (PCy_3_) phosphines as ligands for the Ni catalyst resulted in poor yields (Table 2, entries 2 and 3). Notably, we found that 3 equiv of LiCl was an effective additive for shortening the reaction time (3 h) and improving the yield (73%, Table 2, entry 4) [34]. Other lithium halides, except for LiF, were also effective (Table 2, entries 5–7). However, utilization of Na^+^ and K^+^ as counter cations for the additive provided inferior results (Table 2, entries 8 and 9). Interestingly, switching the counter cation of the base to Li^+^ did not afford any product (Table 2, entry 10). The reaction with PdCl_2_(dppf) instead of NiCl_2_(dppf) reduced the outcome of the reaction (Table 2, entry 11). No product was obtained when boronic acid pinacol ester **6** and borate salt **7** were used as a coupling partner (Table 2, entries 12 and 13) [35]. Dimethoxyethane (DME) and 1,4-dioxane as ethereal solvents did not improve the yields (Table 2, entries 14 and 15). Decreasing the temperature to 80 °C or increasing to 160 °C by microwave irradiation were not effective for improving the reaction (Table 2, entries 16 and 17). Consequently, we found that the reaction shown in entry 4 afforded the optimal result (see Table S1 in Supporting Information File 1 for further manipulation of the reaction conditions).

**Table 2:** Screening of reaction conditions of Suzuki–Miyaura coupling with **3aa**.

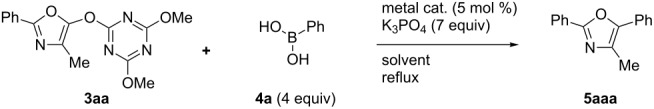						

entry	metal cat.	base	additive (3 equiv)	solvent	time (h)	yield of **5aaa** (%)^a^

1	NiCl_2_(dppf)	K_3_PO_4_	–	toluene	19	21
2	NiCl_2_(dppp)	K_3_PO_4_	–	toluene	12	9
3	NiCl_2_(PCy_3_)_2_	K_3_PO_4_	–	toluene	20	12
4	NiCl_2_(dppf)	K_3_PO_4_	LiCl	toluene	3	73 (68%)^b^
5	NiCl_2_(dppf)	K_3_PO_4_	LiF	toluene	21	35
6	NiCl_2_(dppf)	K_3_PO_4_	LiBr	toluene	3	64
7	NiCl_2_(dppf)	K_3_PO_4_	LiI	toluene	3	70
8	NiCl_2_(dppf)	K_3_PO_4_	NaI	toluene	24	0
9	NiCl_2_(dppf)	K_3_PO_4_	KI	toluene	17	51
10	NiCl_2_(dppf)	Li_3_PO_4_	LiCl	toluene	26	0
11	PdCl_2_(dppf) ·CH_2_Cl_2_	K_3_PO_4_	LiCl	toluene	22	0
12^c^	NiCl_2_(dppf)	K_3_PO_4_	LiCl	toluene	20	0
13^d^	NiCl_2_(dppf)	–	LiCl	toluene	12	0
14	NiCl_2_(dppf)	K_3_PO_4_	LiCl	DME	10	0
15	NiCl_2_(dppf)	K_3_PO_4_	LiCl	1,4-dioxane	21	0
16	NiCl_2_(dppf)	K_3_PO_4_	LiCl	toluene 80 °C	23	36
17	NiCl_2_(dppf)	K_3_PO_4_	LiCl	toluene 160 °C (MW)	20 min	30

^a^NMR yield. ^b^Isolated yield. ^c^Boronic acid pinacol ester **6** was used instead of boronic acid **4a**. ^d^Borate **7** was used instead of boronic acid **4a**.
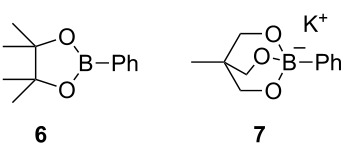

A number of trisubstituted oxazoles were synthesized using 5-(triazinyloxy)oxazoles **3** and various boronic acids **4** (Table 3). To our disappointment, the reaction of **3aa** with the arylboronic acid possessing an electron-withdrawing group **4b** decreased the yield of **5aab** (25%, Table 3, entry 1). Further investigation of the reaction conditions revealed that the reaction with additional dppf (5 mol %) in a sealed tube increased the yield to 64% (Table 3, entry 2) [36–37]. These reaction conditions were defined as conditions B, whereas the conditions in Table 2, entry 4 are defined as conditions A. The arylboronic acid with an electron-donating group **4c** also provided a better yield under conditions B rather than conditions A (Table 3, entries 3 and 4). The desired naphthyloxazole **5aad** was obtained in a high yield (77%, Table 3, entry 5). Introduction of a *p*-tolyl group afforded a good yield of 71% (Table 3, entry 6), whereas reactions of the *o*-tolyl group resulted in moderate yields under both conditions owing to the steric effect (Table 3, entries 7 and 8). The reaction with 3-thienylboronic acid (**4g**) under conditions B proceeded to give **5aag** in 68% yield (Table 3, entries 9 and 10). No product was obtained when *n*-butylboronic acid (**4h**) was used as an aliphatic boronic acid (Table 3, entry 11). Subsequently, the effect of substituents at the 2-position, which was derived from the carboxylic acids, was tested. Aryl substituents possessing both electron-withdrawing and electron-donating groups proceeded to give the corresponding oxazoles in good yields (Table 3, entries 12 and 13). Aliphatic substituents are innocent in the reaction outcome (Table 3, entries 14 and 15). Suzuki–Miyaura coupling of several intermediates **3ab–3af**, which have different substituents at the 4-position, were examined (Table 3, entries 16–20). Compared with **3aa**, the yields using these compounds were slightly lower, especially in the case of sterically more hindered **3ac** and **3af** (Table 3, entries 17 and 20). Thus, Suzuki–Miyaura coupling is affected by steric hindrance from the 4-substituent of oxazoles.

**Table 3:** Synthesis of various trisubstituted oxazoles.

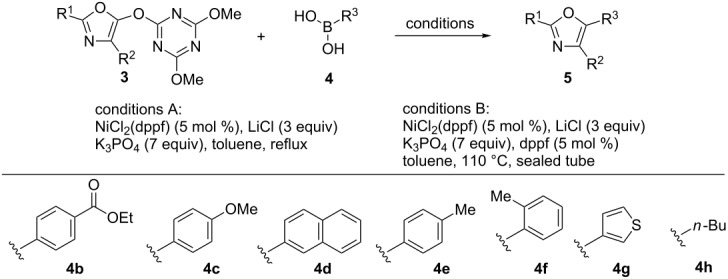					

entry	**3**	**4**	product **5**	conditions	yield of **5** (%)^a^

1	**3aa**	**4b**	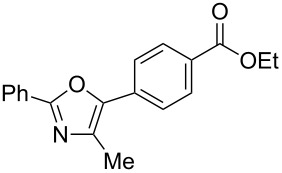 **5aab**	A	25
2	**3aa**	**4b**	**5aab**	B	64
3	**3aa**	**4c**	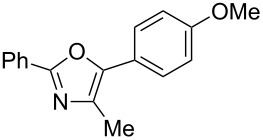 **5aac**	A	54
4	**3aa**	**4c**	**5aac**	B	67
5	**3aa**	**4d**	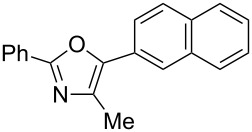 **5aad**	A	77
6	**3aa**	**4e**	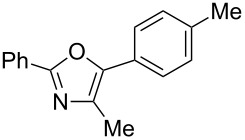 **5aae**	A	71
7	**3aa**	**4f**	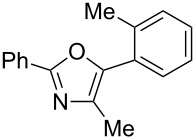 **5aaf**	A	42
8	**3aa**	**4f**	**5aaf**	B	46
9	**3aa**	**4g**	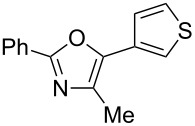 **5aag**	A	30
10	**3aa**	**4g**	**5aag**	B	68
11	**3aa**	**4h**	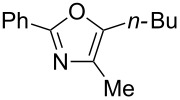 **5aah**	B	0
12	**3ba**	**4a**	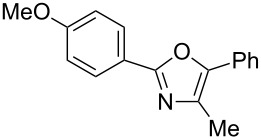 **5baa**	A	69
13	**3ca**	**4a**	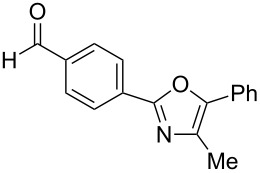 **5caa**	A	64
14	**3da**	**4a**	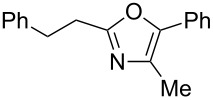 **5daa**	A	68
15	**3ea**	**4a**	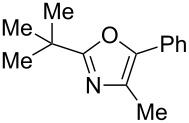 **5eaa**	A	73
16	**3ab**	**4a**	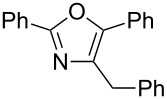 **5aba**	A	60
17	**3ac**	**4a**	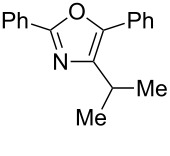 **5aca**	B	54
18	**3ad**	**4a**	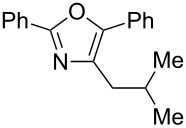 **5ada**	B	62
19	**3ae**	**4a**	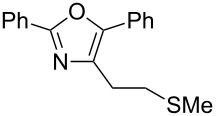 **5aea**	A	62
20	**3af**	**4a**	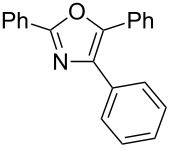 **5afa**	B	47

^a^Isolated yield.

**Scheme 2:**
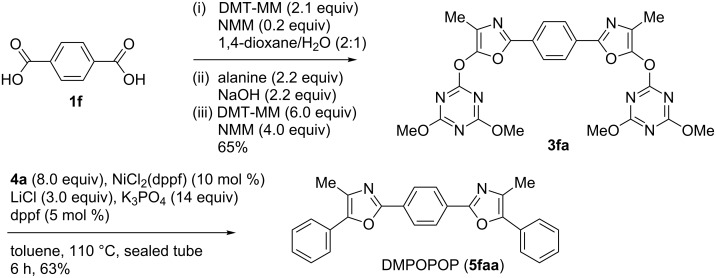
Synthesis of DMPOPOP.

It is noteworthy that the synthesis of bis-oxazole intermediate **3fa** with highly polar terephthalic acid (**1f**) and subsequent double coupling reaction with **4a** successfully proceeded to give DMPOPOP (**5faa**), which is used as a liquid scintillator [38], in a good yield (Scheme 2).

## Conclusion

We have successfully developed a new synthetic method for 2,4,5-trisubstituted oxazoles comprising of carboxylic acids, amino acids, and boronic acids in a one-pot oxazole synthesis with following Ni-catalyzed Suzuki–Miyaura coupling. The combination of various starting materials, which are commercially available, provided the corresponding 2,4,5-trisubstituted oxazoles in good yields. Furthermore, several functionalities, such as ethoxycarbonyl, formyl, and methylsulfanyl groups, which are sensitive to acids, bases, nucleophiles, electrophiles and oxidants, were able to tolerate these reaction conditions (Table 1, entries 3, 9 and Table 3, entries 2, 13 and 19). Therefore, this method is suitable for the synthesis of numerous oxazoles with diverse functionalities.

## Supporting Information

File 1 General information, Table S1, experimental procedure and characterization data for products, and ^1^H and ^13^C NMR spectra.

## References

[1] Turchi I J, Dewar M J S (1975). Chem Rev.

[2] Wipf P (1995). Chem Rev.

[3] Robinson R (1909). J Chem Soc.

[4] Gabriel S (1910). Ber Dtsch Chem Ges.

[5] Davidson D, Weiss M, Jelling M (1937). J Org Chem.

[6] Wipf P, Aoyama Y, Benedum T E (2004). Org Lett.

[7] Patil P C, Luzzio F A, Demuth D R (2015). Tetrahedron Lett.

[8] Bailey J L, Sudini R R (2014). Tetrahedron Lett.

[9] Saito A, Taniguchi A, Kambara Y, Hanzawa Y (2013). Org Lett.

[10] Liu D, Yu J, Cheng J (2014). Tetrahedron.

[11] Zhang L, Zhao X (2015). Org Lett.

[12] Yoshizumi T, Satoh T, Hirano K, Matsuo D, Orita A, Otera J, Miura M (2009). Tetrahedron Lett.

[13] Théveau L, Verrier C, Lassalas P, Martin T, Dupas G, Querolle O, Van Hijfte L, Marsais F, Hoarau C (2011). Chem – Eur J.

[14] Haas D, Mosrin M, Knochel P (2013). Org Lett.

[15] Amaike K, Muto K, Yamaguchi J, Itami K (2012). J Am Chem Soc.

[16] Hodgetts K J, Kershaw M T (2002). Org Lett.

[17] Shaw A Y, Xu Z, Hulme C (2012). Tetrahedron Lett.

[18] Querard P, Girard S A, Uhlig N, Li C-J (2015). Chem Sci.

[19] Kunishima M, Kawachi C, Iwasaki F, Terao K, Tani S (1999). Tetrahedron Lett.

[20] Kunishima M, Kawachi C, Monta J, Terao K, Iwasaki F, Tani S (1999). Tetrahedron.

[21] Kunishima M, Kawachi C, Hioki K, Terao K, Tani S (2001). Tetrahedron.

[22] Kitamura M, Kunishima M, Crich D, Charette A B, Fuchs P L (2013). 4-(4,6-Dimethoxy-1,3,5-triazin-2-yl)-4-methylmorpholinium chloride. e-EROS (Encyclopedia of Reagents for Organic Synthesis) [Online].

[23] Fujita H, Kunishima M (2012). Chem Pharm Bull.

[24] Li X-J, Zhang J-L, Geng Y, Jin Z (2013). J Org Chem.

[25] Iranpoor N, Panahi F (2014). Adv Synth Catal.

[26] Iranpoor N, Panahi F (2015). Org Lett.

[27] Yu B, Sun H, Xie Z, Zhang G, Xu L-W, Zhang W, Gao Z (2015). Org Lett.

[28] Yamada K, Fujita H, Kunishima M (2012). Org Lett.

[29] Yamada K, Yoshida S, Fujita H, Kitamura M, Kunishima M (2015). Eur J Org Chem.

[30] Fujita H, Hayakawa N, Kunishima M (2015). J Org Chem.

[31] Yamada K, Fujita H, Kitamura M, Kunishima M (2013). Synthesis.

[32] Yamada K, Hayakawa N, Fujita H, Kitamura M, Kunishima M (2016). Eur J Org Chem.

[33] Yamada K, Hayakawa N, Fujita H, Kitamura M, Kunishima M (2017). Chem Pharm Bull.

[34] Scott W J, Crisp G T, Stille J K (1984). J Am Chem Soc.

[35] Yamamoto Y, Takizawa M, Yu X-Q, Miyaura N (2008). Angew Chem, Int Ed.

[36] Saito S, Oh-tani S, Miyaura N (1997). J Org Chem.

[R37] 37The reaction of **3aa** and **4a** under conditions B afforded a comparable yield (65%, Table S1, entry 16).

[38] Nemchenok I B, Babin V I, Brudanin V B, Kochetov O I, Timkin V V (2011). Phys Part Nucl Lett.

