# Supplementary material for: Development of a method for the synthesis of 2,4,5-trisubstituted oxazoles composed of carboxylic acid, amino acid, and boronic acid
Source: Beilstein J Org Chem. 2017 Jul 27;13:1478–85. doi: 10.3762/bjoc.13.146 (PMC5550800; doi:10.3762/bjoc.13.146)

Supporting Information

for

Development of a method for the synthesis of 2,4,5-trisubstituted oxazoles composed of carboxylic acid, amino acid, and boronic acid

Kohei Yamada, Naoto Kamimura and Munetaka Kunishima*

Address: Faculty of Pharmaceutical Sciences, Institute of Medical, Pharmaceutical, and Health Sciences, Kanazawa University, Kakuma-machi, Kanazawa 920-1192, Japan

Email: Munetaka Kunishima* - kunisima@p.kanazawa-u.ac.jp

*Corresponding author

General information, Table S1, experimental procedure and characterization data for products, and ^{1}H and ^{13}C NMR spectra

Table of Contents

1. General methods S2
2. Table S1 S3
3. Experimental procedure and characterization data for products S4
4. References S11
5. ^{1}H and ^{13}C NMR spectra S12

1. General methods

Nuclear magnetic resonance (^{1}H NMR (400 MHz or 600 MHz), ^{13}C NMR (100 MHz or 125 MHz)) spectra were determined on a JEOL JNM-ECS400 spectrometer and JEOL JNM-ECS600 spectrometer. Chemical shifts for ^{1}H NMR are reported as δ values relative to tetramethylsilane as the internal standard and coupling constants are in hertz (Hz). The following abbreviations are used for spin multiplicity: s = singlet, d = doublet, t = triplet, m = multiplet, br = broad. Chemical shifts for ^{13}C NMR were reported in ppm relative to the center line of a triplet at 77.16 ppm for deuteriochloroform. NMR yields were determined by quantitative NMR spectroscopy using 1,3,5-trimethoxybenzene, coumarin or 6-methylcoumarin as the internal standard. Mass spectra were measured on a JMS-T100TD AccuTOF TLC (DART-MS) and JMS-SX102A (FAB). Melting points were determined with a Yanagimoto melting point apparatus and are uncorrected. Analytical thin layer chromatography (TLC) was performed on Merck precoated analytical plates, 0.25 mm thick, silica gel 60 F_{254}. Flash chromatography separations were performed on KANTO CHEMICAL Silica Gel 60 N (spherical, neutral, 40-100 mesh).

Reagents and solvents were purchased from Tokyo Chemical Industry (TCI), Nacalai Tesque, Aldrich, and Wako Pure Chemical Industries and were used without further purification unless otherwise noted. Dehydrated 1,4-dioxane, 1,2-dimethoxyethane, toluene and xylene were purchased from commercial sources and distilled over metal sodium before use. All reactions sensitive to oxygen or moisture were conducted under a N_2 atmosphere.

2. Table S1. The results of experiments for Suzuki-Miyaura coupling under other conditions.

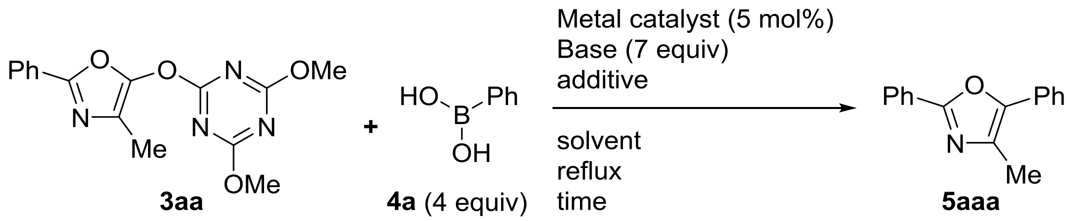

entry	metal cat.	base	additive	solvent	time	yield[a]
1	$NiCl_2$(dppf)	K_3PO_4 (4 equiv)	**4a** (2 equiv)	toluene (0.10 M)	36 h	6
2	$NiCl_2$(dppf)	K_3PO_4	LiCl (3 equiv) **4a** (7 equiv)	toluene	3 h	66
3	$NiCl_2$(dppf)	K_3PO_4	LiCl (1 equiv)	toluene	24 h	31
4	$NiCl_2$(dppf)	K_3PO_4	LiCl (2 equiv)	toluene	24 h	49
5	$NiCl_2$(dppf)	K_3PO_4	LiCl (4 equiv)	toluene	3 h	65
6	$NiCl_2$(dppf)	K_3PO_4	LiCl (5 equiv)	toluene	24 h	36
7	$NiCl_2$(dppf) (10 mol%)	K_3PO_4	LiCl (3 equiv)	toluene	3 h	67
8	$NiCl_2$(dppf)	K_3PO_4	LiI (3 equiv) CuI (5 mol %)	toluene	20 h	0
9	$NiCl_2$(dppf)	K_3PO_4	CuI (3 equiv)	toluene	19 h	0
10	$NiCl_2$(dppf)	K_3PO_4	LiCl (3 equiv) Bu_4NI (0.5 equiv)	toluene	20 h	5
11	$NiCl_2$(dppf)	K_3PO_4	CuI (3 equiv)	toluene	19 h	0
12	$NiCl_2$(dppf)	Cs_2CO_3	LiCl (3 equiv)	toluene	19 h	20
13	$NiCl_2$(dppf)	Li_2CO_3	LiCl (3 equiv)	toluene	26 h	0
14	$NiCl_2$(dppf)	K_3PO_4	LiCl (3 equiv)	toluene 200°C (microwave)	5 min	32
15	$NiCl_2$(dppf)	K_3PO_4	LiCl (3 equiv)	xylene 139 °C	4 h	53
16[b]	$NiCl_2$(dppf)	K_3PO_4	LiCl (3 equiv) dppf (5 mol %)	toluene	4 h	65

[a]NMR yied.
[b]The reaction was conducted using sealed tube.

3. Experimental procedure and characterization data for products

General procedure for the preparataion of (3aa):

To a solution of benzoic acid **1a** (2.2 g, 18.0 mmol) and *N*-methylmorpholine (396 μL, 3.60 mmol) in 1,4-dioxane/H_2O (100 mL:50 mL) was added DMT-MM (5.23 g, 18.9 mmol) at room temperature. After stirring for 15 min, a solution of alanine **2a** (1.76 g, 19.8 mmol) and aq 1 M NaOH (19.8 mL, 19.8 mmol) was added. After the reaction was completed (monitored by TLC), *N*-methylmorpholine (3.96 mL, 36.0 mmol) and DMT-MM (14.9 g, 54 mmol) were added in order. After stirring for 3 h, the reaction mixture was diluted in EtOAc (100 mL) and washed with aq 1 M HCl (40 mL), sat. aq $NaHCO_3$ (40 mL), and brine (30 mL). The organic layer was dried over Na_2SO_4, filtered, and evaporated under reduced pressure. The residue was purified by column chromatography (EtOAc:hexane = 7:3) to give oxazole **3aa** in 78% yield.

5-((4,6-Dimethoxy-1,3,5-triazin-2-yl)oxy)-4-methyl-2-phenyloxazole (3aa)[1]

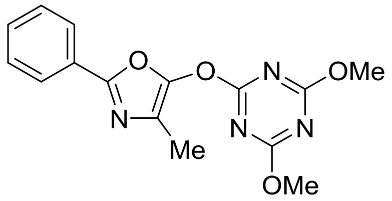

Yield: 78%. White solid. ^{1}H NMR (400MHz, $CDCl_3$): δ 7.98-7.92 (m, 2H), 7.47-7.40 (m, 3H), 4.03 (s, 6H), 2.15 (s, 3H); ^{13}C NMR (100 MHz, $CDCl_3$): δ 174.1, 172.6, 154.9, 146.4, 130.2, 128.8, 127.3, 125.8, 120.6, 55.9, 10.4; HRMS (DART+) Calcd for $C_{15}H_{15}N_4O_4^+$ ($[M+H]^+$): 315.1093, Found: 315.1093.

5-((4,6-Dimethoxy-1,3,5-triazin-2-yl)oxy)-2-(4-methoxyphenyl)-4-methyloxazole (3ba)

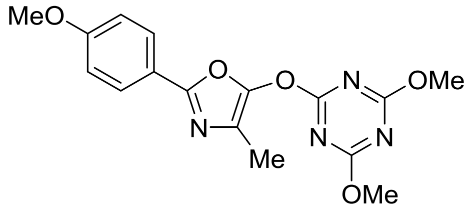

Yield: 69%. Colorless crystal (recrystallization from CH_2Cl_2 and hexane). mp: 164-166 °C. ^{1}H NMR (400MHz, $CDCl_3$): δ 7.88 (d, J = 8.2 Hz, 2H), 6.94 (d, J = 8.2 Hz, 2H), 4.03 (s, 6H), 3.85 (s, 3H), 2.13 (s, 3H); ^{13}C NMR (100 MHz, $CDCl_3$): δ 174.2, 172.8, 161.4, 155.2, 146.1, 127.6, 120.3, 120.2, 114.3, 56.0, 55.5, 10.5; HRMS (DART+) Calcd for $C_{16}H_{17}N_4O_5^+$ ($[M+H]^+$): 345.1199, Found: 345.1202 ; Anal. Calcd for $C_{16}H_{16}N_4O_5$: C, 55.81; H, 4.68; N, 16.27. Found: C, 55.78; H, 4.64; N, 16.46.

5-(5-((4,6-Dimethoxy-1,3,5-triazin-2-yl)oxy)-2-(4-formylphenyl)-4-methyloxazole (3ca)

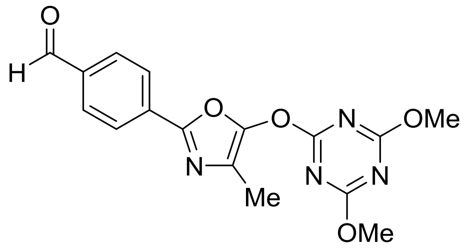

Yield: 60%. Colorless crystal (recrystallization from EtOAc and hexane). mp: 109-111 °C. ^{1}H NMR (600 MHz, $CDCl_3$): δ 10.06 (s, 1H), 8.11 (d, J = 8.2 Hz, 2H), 7.95 (d, J = 8.2 Hz, 2H), 4.05 (s, 6H), 2.18 (s, 3H); ^{13}C NMR (150 MHz, $CDCl_3$): δ 191.6, 174.2, 172.6, 153.7, 147.3, 137.2, 132.4, 130.2, 126.3, 121.9, 56.1, 10.5; HRMS (DART+) Calcd for $C_{16}H_{15}N_4O_5^+$ ($[M+H]^+$): 343.1042, Found: 343.1023; Anal. Calcd for $C_{16}H_{14}N_4O_5$: C, 56.14; H, 4.12; N, 16.37. Found: C, 55.89; H, 4.17; N, 16.48.

5-((4,6-Dimethoxy-1,3,5-triazin-2-yl)oxy)-4-methyl-2-phenethyloxazole (3da)

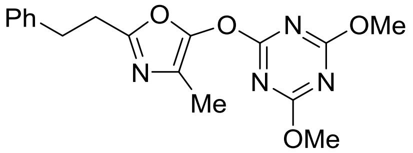

Yield: 54%. Colorless oil. ^{1}H NMR (600 MHz, $CDCl_3$): δ 7.31-7.25 (m, 2H), 7.23-7.18 (m, 3H), 4.02 (s, 6H), 3.09-3.04 (m, 2H), 3.01-2.96 (m, 2H), 2.04 (s, 3H); ^{13}C NMR (150 MHz, $CDCl_3$): δ 174.1, 172.6, 157.4, 146.0, 140.3, 128.6, 128.4, 126.4, 118.7, 55.9, 33.0, 30.4, 10.2; HRMS (DART+) Calcd for $C_{17}H_{19}N_4O_4^+$ ($[M+H]^+$): 343.1406, Found: 343.1392; Anal. Calcd for $C_{17}H_{18}N_4O_4$: C, 59.64; H, 5.30; N, 16.37. Found: C, 59.99; H, 5.46; N, 16.28.

2-(*tert*-Butyl)-5-((4,6-dimethoxy-1,3,5-triazin-2-yl)oxy)-4-methyloxazole (3ea)

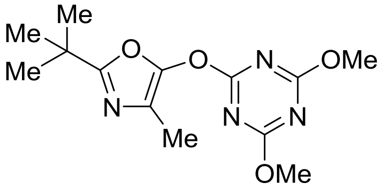

Yield: 71%. White solid. ^{1}H NMR (400 MHz, $CDCl_3$): δ 4.02 (s, 6H), 2.05 (s, 3H), 1.36 (d, 9H); ^{13}C NMR (100 MHz, $CDCl_3$): δ 174.2, 172.7, 164.5, 145.8, 118.1, 55.9, 33.9, 28.4, 10.4; HRMS (DART+) Calcd for $C_{13}H_{19}N_4O_4^+$ ($[M+H]^+$): 295.1406, Found: 295.1400; Anal. Calcd for $C_{13}H_{18}N_4O_4$: C, 53.05; H, 6.16; N,19.04. Found: C, 52.90; H, 6.21; N, 19.07.

1,4-Bis(5-((4,6-dimethoxy-1,3,5-triazin-2-yl)oxy)-4-methyloxazol-2-yl)benzene (3fa)

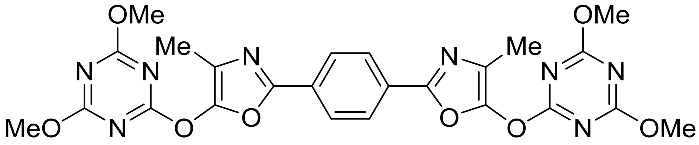

Yield: 65%. White solid. ^{1}H NMR (400 MHz, $CDCl_3$): δ 8.01 (s, 4H), 4.04 (s, 12H), 2.16 (s, 6H); ^{13}C NMR (100 MHz, $CDCl_3$): δ 174.2, 172.7, 154.3, 146.8, 128.6, 126.3, 121.3, 56.1, 10.6. HRMS (FAB+) Calcd for $C_{24}H_{23}N_8O_8^+$ ($[M+H]^+$): 551.1639, Found: 551.1633.

4-Benzyl-5-((4,6-dimethoxy-1,3,5-triazin-2-yl)oxy)-2-phenyloxazole (3ab)

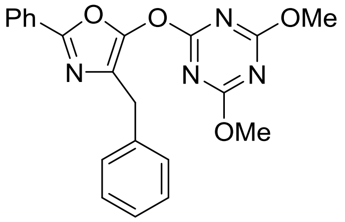

Yield: 78%. Colorless oil. ^{1}H NMR (600 MHz, $CDCl_3$): δ 7.98-7.93 (m, 2H), 7.44-7.40 (m, 3H), 7.28-7.24 (m, 2H), 7.23-7.19 (m, 2H), 7.17-7.13 (m, 1H), 3.96 (s, 6H), 3.88 (s, 2H); ^{13}C NMR (100 MHz, $CDCl_3$): δ 174.0, 172.6, 155.3, 147.0, 137.7, 130.4, 129.0, 128.8, 128.4, 127.4, 126.5, 126.1, 123.5, 56.0, 31.6; HRMS (DART+) Calcd for $C_{21}H_{19}N_4O_4^+$ ($[M+H]^+$): 391.1406, Found: 391.1383; Anal. Calcd for $C_{21}H_{18}N_4O_4$: C, 64.61; H, 4.65; N,14.35, Found: C, 64.48; H, 4.86; N, 14.03.

5-((4,6-Dimethoxy-1,3,5-triazin-2-yl)oxy)-4-isopropyl-2-phenyloxazole (3ac)

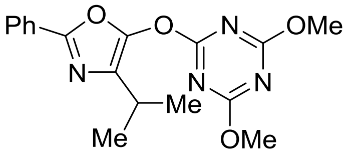

Yield: 83%. Colorless oil. ^{1}H NMR ($CDCl_3$): δ 7.98-7.92 (m, 2H), 7.45-7.39 (m, 3H), 4.02 (s, 6H), 2.89 (sep, J = 6.9 Hz, 1H), 1.27 (d, J = 6.9 Hz, 6H); ^{13}C NMR ($CDCl_3$): δ 174.2, 173.1, 155.0, 145.0, 130.2, 129.8, 128.8, 127.6, 126.0, 56.0,

25.6, 21.2; HRMS (DART+) Calcd for $C_{17}H_{19}N_4O_4^+$ ($[M+H]^+$): 343.1406, Found: 343.1406; Anal. Calcd for $C_{17}H_{18}N_4O_4$: C, 59.64; H, 5.30; N, 16.37. Found: C, 59.32; H, 5.49; N, 16.08.

5-((4,6-Dimethoxy-1,3,5-triazin-2-yl)oxy)-4-isobutyl-2-phenyloxazole (3ad)[1]

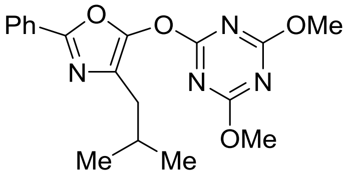

Yield: 70%. Colorless oil. ^{1}H NMR (600MHz, $CDCl_3$): δ 7.98-7.93 (m, 2H), 7.46-7.40 (m, 3H), 4.02 (s, 6H), 2.36 (d, J = 7.2 Hz, 2H), 2.07 (m, 1H), 0.95 (d, J = 6.5 Hz, 6H); ^{13}C NMR (150MHz, $CDCl_3$): δ 174.1, 172.9, 155.0, 146.9, 130.2, 128.8, 127.5, 125.9, 124.1, 55.9, 33.9, 27.7, 22.4; HRMS (ESI+) Calcd for $C_{18}H_{21}N_4O_4$ ($[M-H]^+$): 357.1563, Found: 357.1580.

5-((4,6-Dimethoxy-1,3,5-triazin-2-yl)oxy)-4-(2-(methylthio)ethyl)-2-phenyloxazole (3ae)

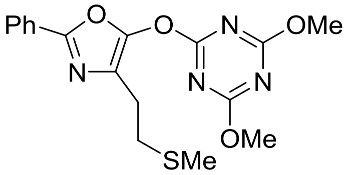

Yield: 70%. White solid. ^{1}H NMR (600 MHz, $CDCl_3$): δ 7.98-7.92 (m, 2H), 7.45-7.41 (m, 3H), 4.03 (s, 6H), 2.86-2.81 (m, 2H), 2.82-2.77 (m, 2H), 2.12 (s, 3H); ^{13}C NMR (150 Hz, $CDCl_3$): δ 174.8, 172.8, 155.3, 146.7, 130.4, 128.9, 127.3, 126.0, 123.2, 56.0, 32.4, 25.5, 15.6; HRMS (DART+) Calcd for $C_{17}H_{19}N_4O_4S^+$ ($[M+H]^+$): 375.1127, Found: 375.1105; Anal. Calcd for $C_{17}H_{18}N_4O_4S$: C, 54.53; H, 4.85; N,14.96. Found: C, 54.46; H, 4.91; N, 14.86.

5-((4,6-Dimethoxy-1,3,5-triazin-2-yl)oxy)-2,4-diphenyloxazole (3af)[1]

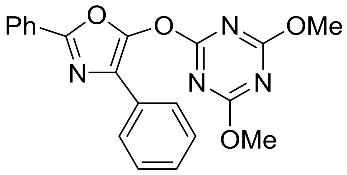

Yield: 78%. Yellow solid. ^{1}H NMR (600 MHz, $CDCl_3$): δ 8.09-8.02 (m, 2H), 7.85-7.81 (m, 2H), 7.50-7.45 (m, 3H), 7.43-7.37 (m, 2H), 7.32-7.27 (m, 1H), 3.97 (s, 6H); ^{13}C NMR (150 MHz, $CDCl_3$): δ 174.1, 172.5, 155.2, 145.8, 130.5, 130.0, 128.8, 128.7, 128.0, 127.8, 127.1, 126.1, 126.0, 125.8, 125.6, 123.8, 55.9; HRMS (DART+) Calcd for $C_{20}H_{17}N_4O_4^+$ ($[M+H]^+$): 377.1250, Found: 377.1238.

General procedure for the synthesis of 5

Conditions A: To a round-bottom flask equiped with a reflux condenser were added 5-(triazinyloxy)oxazole **3aa** (100 mg, 0.318 mmol), phenylboronic acid (**4a**, 155 mg, 1.27 mmol), $NiCl_2$(dppf) (10.9 mg, 0.318 mmol), K_3PO_4 (473 mg, 2.23 mmol), LiCl (40.4 mg, 0.954 mmol) and toluene (2.3 mL) in N_2. After the reaction mixture was stirred at 110 °C for 3 h, the reaction mixture was cooled to room temperature. The reaction mixture was diluted in CH_2Cl_2 (10 mL), filtred through a short pad of Celite, and the filtrate was concentrated under reduced pressure. The residue was purified by flash column chromatography (CH_2Cl_2:hexane = 70:30) to give **5aaa** (68%) as a white solid.

Conditions B: To a screw-capped tube was added 5-(triazinyloxy)oxazole **3aa** (100 mg, 0.318 mmol), 4-ethoxycarbonylphenylboronic acid (**4b**, 246 mg, 1.27 mmol), $NiCl_2$(dppf) (10.9 mg, 0.0159 mmol), K_3PO_4 (473 mg, 2.23 mmol), LiCl (40.4 mg, 0.954 mmol), DPPF (8.8 mg, 0.0159 mmol) and toluene (2.3 mL) in N_2. After the mixture was stirred at 110 °C for 6 h, the reaction mixture was cooled to room temperature. The reaction mixture was diluted in CH_2Cl_2 (10 mL), filtred through a short pad of Celite, and the filtrate was concentrated under reduced pressure. The residue was purified by flash column chromatography (EtOAc:hexane = 10:90) to give **5aab** (62.5 mg, 64%) as a white solid.

4-Methyl-2,5-diphenyloxazole (5aaa)[2]

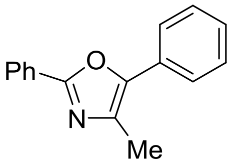

Synthesized under conditions A in 68% yield. White solid. ^{1}H NMR (400 MHz, $CDCl_3$): δ 8.12-8.06 (m, 2H), 7.72-7.66 (m, 2H), 7.50-7.41 (m, 5H), 7.37-7.30 (m, 1H), 2.51 (s, 3H); ^{13}C NMR (100 MHz, $CDCl_3$): δ 159.5, 145.6, 133.5, 130.3, 129.3, 128.94, 128.91, 127.8, 127.6, 126.4, 125.5, 13.7; HRMS (DART+) Calcd for $C_{16}H_{14}NO^+$ ($[M+H]^+$): 236.1075, Found: 236.1065.

4-Methyl-2-phenyl-5-(4-ethoxycarbonylphenyl)oxazole (5aab)

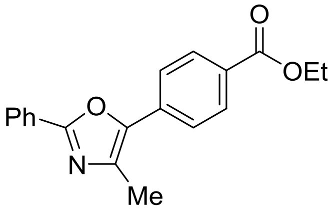

Synthesized under conditions B in 64% yield (6 h). White solid. ^{1}H NMR (600 MHz, $CDCl_3$): δ 8.15-8.11 (m, 2H), 8.11-8.07 (m, 2H), 7.77-7.72 (m, 2H), 7.51-7.43 (m, 3H), 4.41 (q, J = 7.2 Hz, 2H), 2.54 (s, 3H), 1.42 (t, J = 7.2 Hz, 3H); ^{13}C NMR (150 MHz, $CDCl_3$): δ 166.3, 160.2, 144.8, 135.7, 133.3, 130.6, 130.2, 129.2, 129.0, 127.3, 126.5, 124.8, 61.2, 14.5, 14.0; HRMS (DART+) Calcd for $C_{19}H_{18}NO_3$ ($[M+H]^+$): 308.1287, Found: 308.1297; Anal. Calcd for $C_{19}H_{17}NO_3$: C, 74.25; H, 5.58; N, 4.56. Found: C, 74.27; H, 5.70; N, 4.45.

4-Methyl-5-(4-methoxyphenyl)-2-phenyloxazole (5aac)[3]

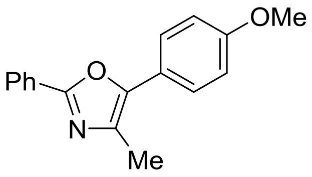

Synthesized under conditions B in 67% yield (6 h). White solid. ^{1}H NMR (600 MHz, $CDCl_3$): δ 8.09-8.04 (m, 2H), 7.63-7.58 (m, 2H), 7.48-7.39 (m, 3H), 7.02-6.97 (m, 2H), 3.85 (s, 3H), 2.46 (s, 3H); ^{13}C NMR (150 MHz, $CDCl_3$): δ 159.3, 159.0, 145.7, 132.0, 130.1, 128.9, 127.7, 127.0, 126.2, 122.1, 144.4, 55.5, 13.5; HRMS (DART+) Calcd for $C_{17}H_{16}NO_2$ ($[M+H]^+$): 266.1181, Found: 266.1205.

4-Methyl-5-(2-naphtyl)-2-phenyloxazole (5aad)

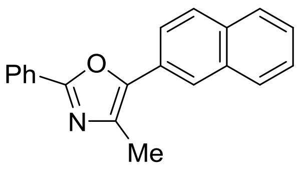

Synthesized under conditions A in 77% yield (1 h). White solid. ^{1}H NMR (600 MHz, $CDCl_3$): δ 8.16-8.09 (m, 3H), 7.94-7.88 (m, 2H), 7.87-7.80 (m, 2H), 7.55-7.43 (m, 5H), 2.59 (s, 3H); ^{13}C NMR (150 MHz, $CDCl_3$): δ 159.7, 145.8, 134.0, 133.5, 132.7, 130.4, 128.9, 128.7, 128.3, 127.9, 127.6, 126.8, 126.7, 126.5, 126.4, 124.4, 123.3, 13.9; HRMS (DART+) Calcd for $C_{20}H_{16}NO$ ($[M+H]^+$): 286.1232, Found: 286.1231; Anal. Calcd for $C_{20}H_{15}NO$: C, 84.19; H, 5.30; N, 4.91. Found: C, 83.95; H, 5.36; N, 4.87.

4-Methyl-2-phenyl-5-(4-tolyl)oxazole (5aae)[3]

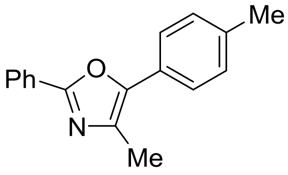

Synthesized under conditions A in 71% yield (6 h). White solid. ^{1}H NMR (600 MHz, $CDCl_3$): δ 8.10-8.06 (m, 2H), 7.60-7.56 (m, 2H), 7.49-7.41 (m, 3H), 7.30-7.25 (m, 2H), 2.48 (s, 3H), 2.40 (s, 3H); ^{13}C NMR (150 MHz, $CDCl_3$): δ 159.2, 145.8, 137.7, 132.8, 130.2, 129.6, 128.9, 127.7, 126.5, 126.3, 125.5, 21.5, 13.6; HRMS (DART+) Calcd for $C_{17}H_{16}NO$ ($[M+H]^+$): 250.1232, Found: 250.1243.

4-Methyl-2-phenyl-5-(2-tolyl)oxazole (5aaf)

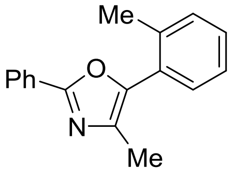

Synthesized under conditions B in 46% yield (6 h). colorless oil. ^{1}H NMR (600 MHz, $CDCl_3$): δ 8.08-8.03 (m, 2H), 7.48-7.40 (m, 3H), 7.40-7.36 (m, 1H), 7.35-7.25 (m, 3H), 2.42 (s, 3H), 2.29 (s, 3H); ^{13}C NMR (150 MHz, $CDCl_3$): δ 160.2, 146.2, 137.6, 134.3, 131.0, 130.2, 129.9, 129.1, 128.9, 128.2, 127.8, 126.2, 125.9, 20.7, 12.6; HRMS (DART+) Calcd for $C_{17}H_{16}NO$ ($[M+H]^+$): 250.1232, Found: 250.1251; Anal. Calcd for $C_{17}H_{15}NO$: C, 81.90; H, 6.06; N, 5.62. Found: C, 82.13; H, 6.27; N, 5.45.

4-Methyl-2-phenyl-5-(3-thienyl)oxazole (5aag)

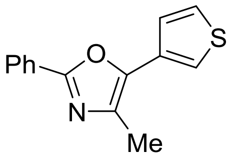

Synthesized under conditions B in 68% yield (6 h). White solid. ^{1}H NMR (600 MHz, $CDCl_3$): δ 8.08-8.04 3(m, 2H), 7.51-7.47 (m, 1H), 7.47-7.39 (m, 5H), 2.44 (s, 3H); ^{13}C NMR (150 MHz, $CDCl_3$): δ 159.0, 143.0, 132.5, 130.2, 130.1, 128.9, 127.5, 126.6, 126.2, 125.2, 120.6, 13.1; HRMS (DART+) Calcd for $C_{14}H_{12}NOS$ ($[M+H]^+$): 242.0640, Found: 242.0668; Anal. Calcd for $C_{14}H_{11}NOS$: C, 69.68; H, 4.59; N, 5.80. Found: C, 69.73; H, 4.79; N, 5.61.

4-Methyl-2-(4-methoxyphenyl)-5-phenyloxazole (5baa)[4]

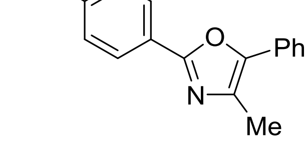

Synthesized under conditions A in 69% yield (4 h). White solid. ^{1}H NMR (400 MHz, $CDCl_3$): δ 8.04-7.99 (m, 2H), 7.69-7.64 (m, 2H), 7.49-7.42 (m, 2H), 7.34-7.28 (m, 1H), 7.00-6.95 (m, 2H), 3.86 (s, 3H), 2.48 (s, 3H); ^{13}C NMR (100 MHz, $CDCl_3$): δ 161.4, 159.6, 145.0, 133.3, 129.5, 128.9, 128.0, 127.5, 125.3, 120.4, 114.3, 55.5, 13.7; HRMS (DART+) Calcd for $C_{17}H_{16}NO_2^+$ ($[M+H]^+$): 266.1181, Found: 266.1175.

2-(4-Formylphenyl)-4-methyl-5-phenyloxazole (5caa)

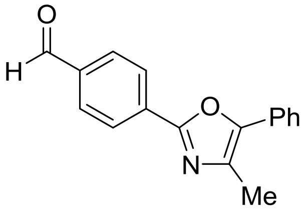

Synthesized under conditions A in 64% yield (4 h). White solid. ^{1}H NMR (400 MHz, $CDCl_3$): δ 10.1 (s, 1H), 8.28-8.22 (m, 2H), 8.01-7.96 (m, 2H), 7.74-7.68 (m, 2H), 7.53-7.46 (m, 2H) 7.41-7.34 (m, 1H), 2.53 (s, 3H); ^{13}C NMR (100 MHz, $CDCl_3$): δ 191.7, 158.1, 146.8, 137.1, 134.3, 132.6, 130.3, 129.1, 128.8, 128.3, 126.7, 125.7, 13.7; HRMS (DART+) Calcd for $C_{17}H_{14}NO_2^+$ ($[M+H]^+$): 264.1025, Found: 264.1054; Anal. Calcd for $C_{17}H_{13}NO_2$: C, 77.55; H, 4.98; N,5.32. Found: C, 77.50; H, 5.05; N, 5.31.

4-Methyl-2-phenethyl-5-phenyloxazole (5daa)

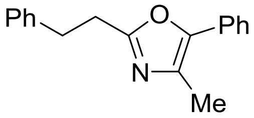

Synthesized under conditions A in 68% (2 h). White solid. ^{1}H NMR (600 MHz, $CDCl_3$): δ 7.58-7.53 (m, 2H), 7.44-7.39 (m, 2H), 7.32-7.27 (m, 3H), 7.26-7.19 (m, 3H), 3.16-3.10 (m, 2H) 3.11-3.05 (m, 2H), 2.40 (s, 3H); ^{13}C NMR (150 MHz, $CDCl_3$): δ 162.0, 145.2, 140.6, 131.7, 129.4, 128.8, 128.7, 128.4, 127.5, 126.5, 125.3, 33.4, 30.2, 13.4; HRMS (DART+) Calcd for $C_{18}H_{18}NO$ ($[M+H]^+$): 264.1388, Found: 264.1395; Anal. Calcd for $C_{18}H_{17}NO$: C, 82.10; H, 6.51; N, 5.32. Found: C, 82.00; H, 6.64; N, 5.28.

4-Methyl-2-*tert*-butyl-5-phenyloxazole (5eaa)

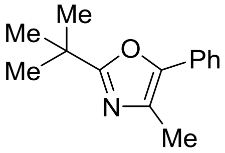

Synthesized under conditions A in 73% yield (4 h). colorless oil. ^{1}H NMR (400 MHz, $CDCl_3$): δ 7.61-7.55 (m, 2H), 7.46-7.39 (m, 2H), 7.32-7.25 (m, 1H), 2.40 (s, 3H), 1.43 (s, 9H); ^{13}C NMR (100 MHz, $CDCl_3$): δ 169.3, 144.7, 131.3, 129.7, 128.8, 127.3, 125.3, 33.7, 28.8, 13.4; HRMS (DART+) Calcd for $C_{13}H_{19}NO^+$ ($[M+H]^+$):216.1388, Found: 216.1387.

2,2'-(1, 4-Phenylene) bis(4-methyl-5-phenyl)oxazole (5faa)

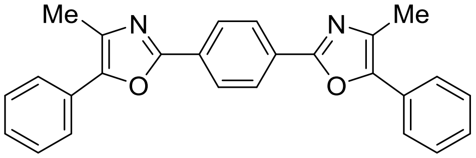

Synthesized under conditions B in 63% yield (6 h). Yellow solid. ^{1}H NMR (600 MHz, $CDCl_3$): δ 8.18 (s, 4H), 7.75-7.68 (m, 4H), 7.52-7.45 (m, 4H), 7.39-7.32 (m, 2H), 2.53 (s, 6H); ^{13}C NMR (150 MHz, $CDCl_3$): δ 158.9, 146.1, 133.9, 129.1, 129.0, 128.7, 128.0, 126.7, 125.6, 13.7. HRMS (FAB+) Calcd for $C_{26}H_{21}N_2O_2^+$ ($[M+H]^+$):393.1603, Found: 393.1591.

4-Benzyl-2,5-diphenyloxazole (5aba)[5]

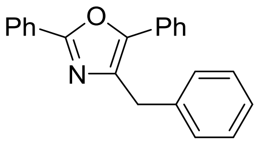

Synthesized under conditions A in 60% (4 h). White solid. ^{1}H NMR (600 MHz, $CDCl_3$): δ 8.13-8.09 (m, 2H), 7.69-7.64 (m, 2H), 7.49-7.40 (m, 5H), 7.36-7.27 (m, 5H), 7.24-7.19 (m, 1H), 4.22 (s, 2H); ^{13}C NMR (150 MHz, $CDCl_3$): δ 160.0, 146.7, 138.7, 135.9, 130.4, 129.0, 128.91, 128.88, 128.7, 128.6, 128.2, 127.6, 126.5, 125.8, 33.3; HRMS (DART+) Calcd for $C_{22}H_{18}NO$ ($[M+H]^+$): 312.1388, Found: 312.1390.

4-Isopropyl-2,5-diphenyloxazole (5aca)[5]

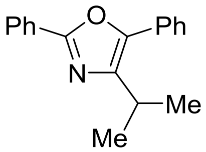

Synthesized under conditions B in 54% yield (6 h). White solid. ^{1}H NMR (600 MHz, $CDCl_3$): δ 8.13-8.07 (m, 2H), 7.68-7.62 (m, 2H), 7.49-7.40 (m, 5H), 7.37-7.31 (m, 1H), 3.29 (m, 1H), 1.38 (d, J = 6.8 Hz, 2H); ^{13}C NMR (150 MHz, $CDCl_3$): δ 159.9, 144.1, 143.5, 130.1, 129.6, 128.9, 128.8, 128.0, 127.8, 126.5, 126.1, 26.2, 22.2; HRMS (DART+) Calcd for $C_{18}H_{18}NO$ ($[M+H]^+$): 264.1388, Found: 264.1394.

4-Isobutyl-2,5-diphenyloxazole (5ada)

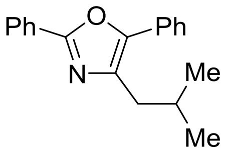

Synthesized under conditions B in 62% yield (6 h). White solid. ^{1}H NMR (600 MHz, $CDCl_3$): δ 8.12-8.07 (m, 2H), 7.72-7.67 (m, 2H), 7.48-7.40 (m, 5H), 7.35-7.30 (m, 1H), 2.71 (d, J = 7.3 Hz, 2H), 2.22 (m, 1H), 1.02 (d, J = 6.5 Hz, 6H); ^{13}C NMR (150 MHz, $CDCl_3$): δ 159.6, 146.0, 137.5, 130.2, 129.5, 128.9, 128.8, 127.81, 127.77, 126.4, 125.8, 36.3, 28.5, 22.7; HRMS (DART+) Calcd for $C_{19}H_{20}NO$ ($[M+H]^+$): 278.1545, Found: 278.1535; Anal. Calcd for $C_{19}H_{19}NO$: C, 82.28; H, 6.90; N, 5.05. Found: C, 82.19; H, 6.93; N, 5.00.

4-(2-(Methylthio)ethyl)-2,5-diphenyloxazole (5aea)

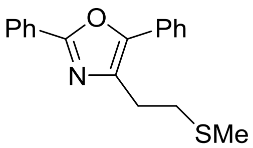

Synthesized under conditions A in 62% yield (4 h). White solid. ^{1}H NMR (600 MHz, $CDCl_3$): δ 8.11-8.06 (m, 2H), 7.72-7.67 (m, 2H), 7.50-7.42 (m, 5H), 7.38-7.33 (m, 1H), 3.15-3.10 (m, 2H), 2.99-2.94 (m, 2H), 2.17 (s, 3H); ^{13}C NMR (150 MHz, $CDCl_3$): δ 159.9, 146.1, 136.1, 130.4, 129.02, 128.99, 128.89, 128.2, 127.6, 126.5, 125.9, 33.1, 27.8, 15.9; HRMS (DART+) Calcd for $C_{18}H_{18}NOS$ ($[M+H]^+$): 296.1109, Found: 296.1110.

2,4,5-Triphenyloxazole (5afa)[6]

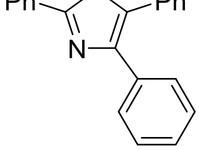

Synthesized under conditions B in 47% yield (6 h). White solid. ^{1}H NMR (600 MHz, $CDCl_3$): δ 8.18-8.13 (m, 2H), 7.75-7.65 (m, 4H), 7.51-7.44 (m, 3H), 7.43-7.31 (m, 6H); ^{13}C NMR (150 MHz, $CDCl_3$): δ 160.3, 145.7, 136.9, 132.7, 130.5, 129.1, 128.9, 128.82, 128.76, 128.69, 128.4, 128.3, 127.5, 126.7, 126.6; HRMS (DART+) Calcd for $C_{21}H_{16}NO$ ($[M+H]^+$): 298.1232, Found: 298.1227

4. References

1) Fujita, H.; Kunishima, M. *Chem. Pharm. Bull.* **2012**, *60*, 907-912
2) Imai, S.; Kikui, H.; Moriyama, K.; Togo, H. *Tetrahedron* **2015**, *71*, 5267-5274.
3) Kumar, M. P.; Liu, R.-S. *J. Org. Chem.* **2006**, *71*, 4951-4955.
4) Zhang, F.; Greaney, M. F. *Org. Lett.* **2010**, *12*, 4745-4747.
5) Keni, M.; Tepe, J. J. *J. Org. Chem.* **2005**, *70*, 4211-4213.
6) Chatterjee, T.; Cho, J. Y.; Cho, E. J. *J. Org. Chem.* **2016**, *81*, 6995-7000.

5. ^{1}H and ^{13}C NMR spectra

5-((4,6-Dimethoxy-1,3,5-triazin-2-yl)oxy)-4-methyl-2-phenyloxazole (3aa)

^{1}H NMR (400 MHz, $CDCl_3$)

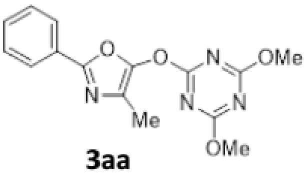

3aa

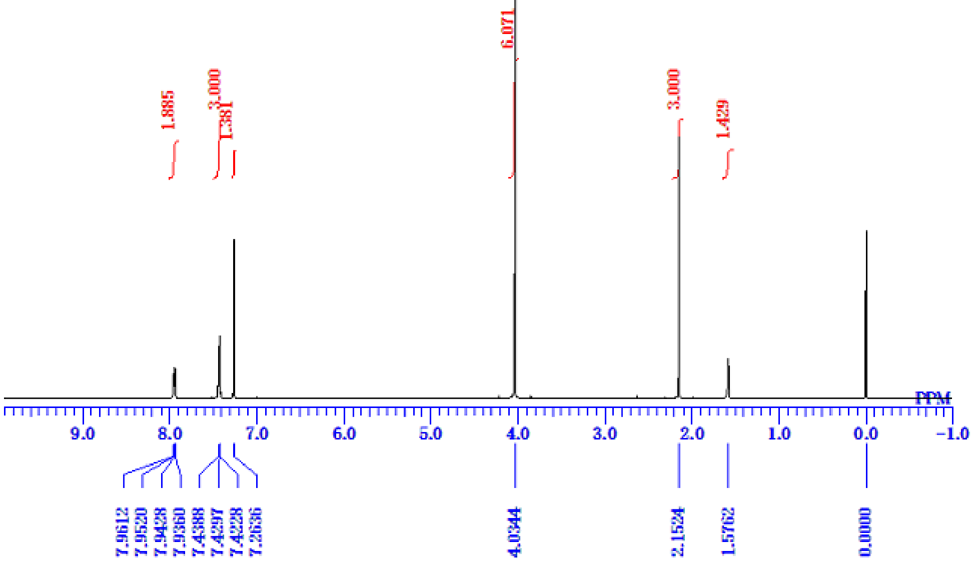

^{13}C NMR (100 MHz, $CDCl_3$)

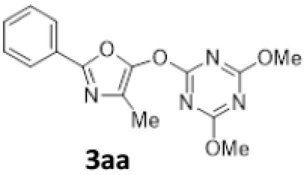

3aa

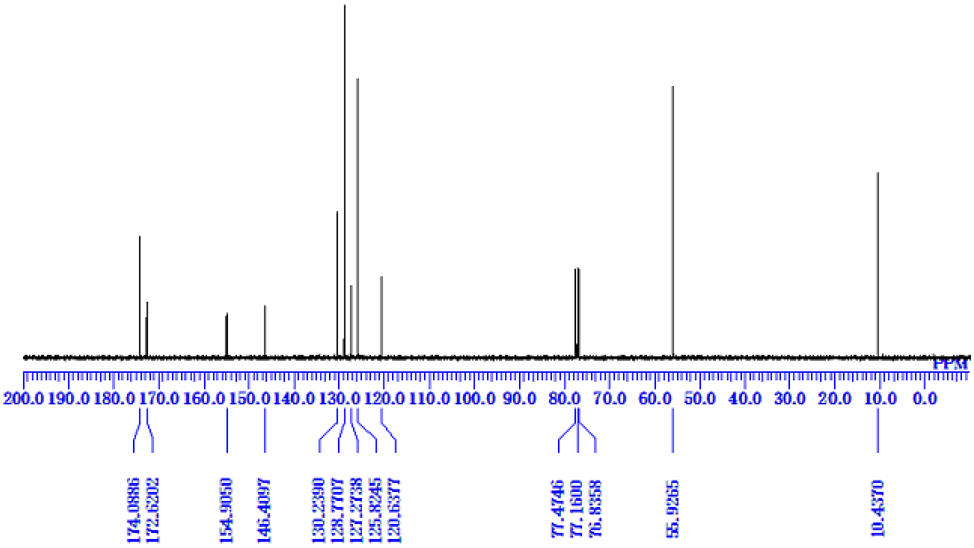

5-((4,6-Dimethoxy-1,3,5-triazin-2-yl)oxy)-2-(4-methoxyphenyl)-4-methyloxazole (3ba)

^{1}H NMR (400 MHz, $CDCl_3$)

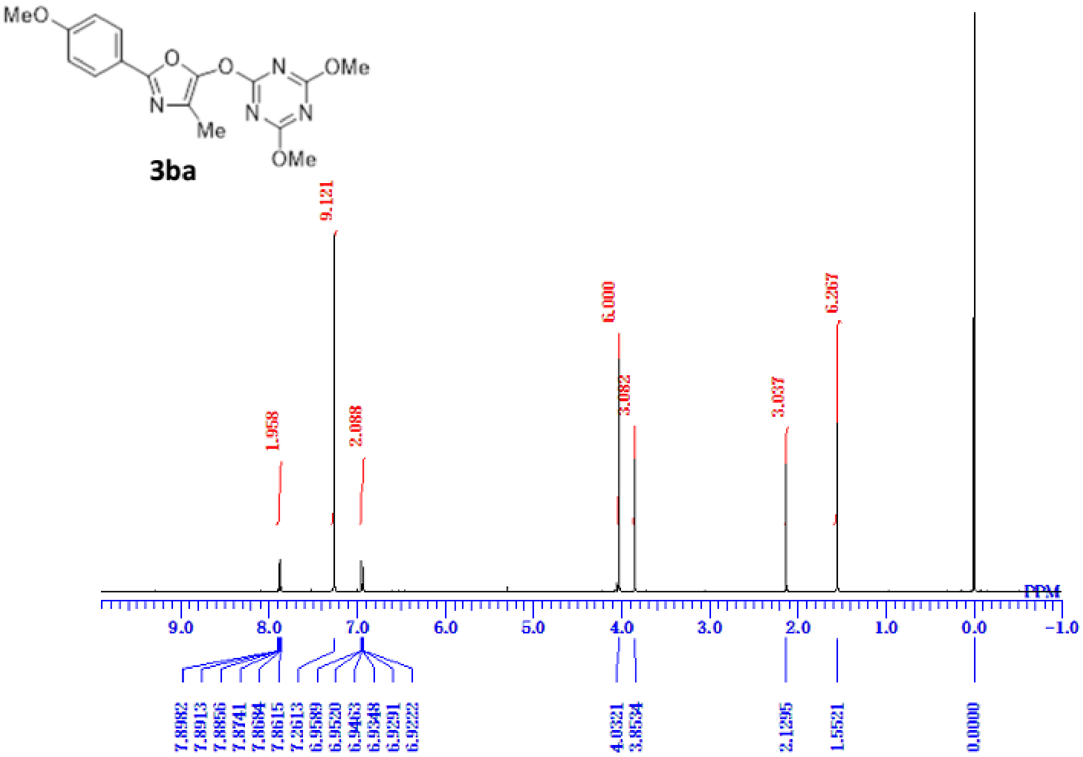

^{13}C NMR (100 MHz, $CDCl_3$)

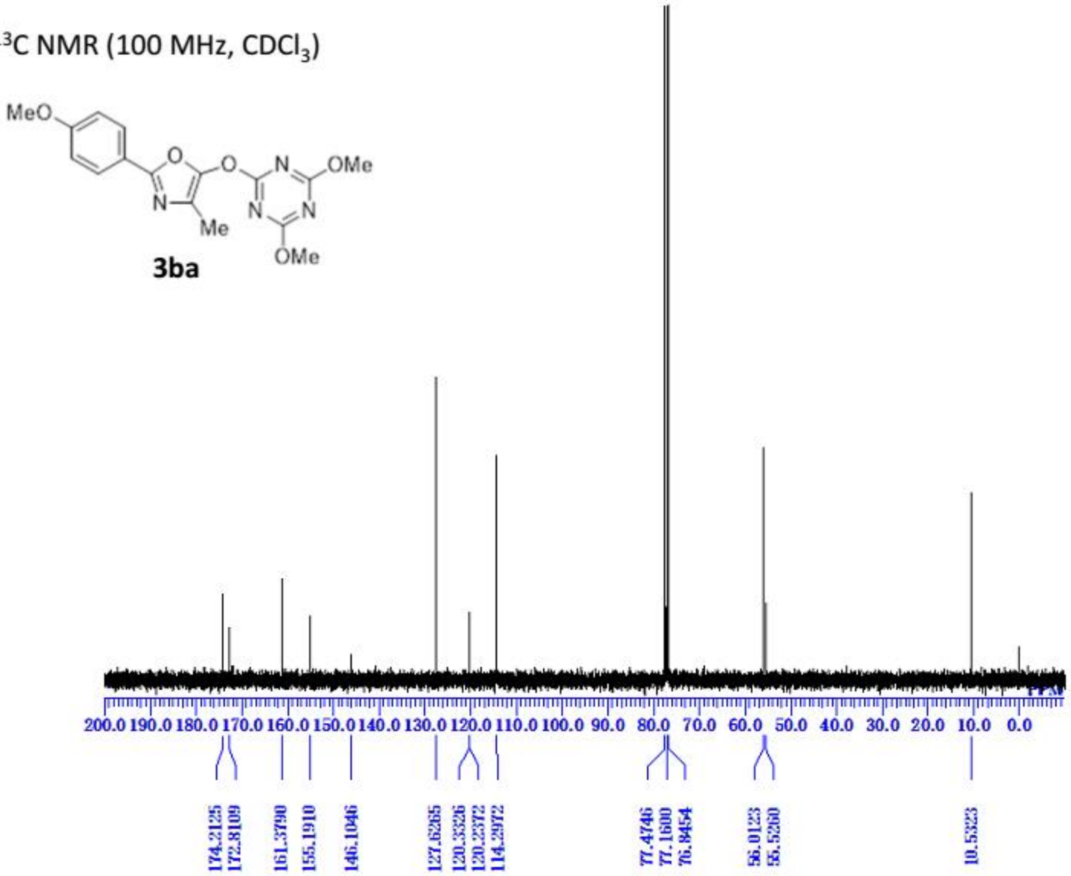

5-(5-((4,6-Dimethoxy-1,3,5-triazin-2-yl)oxy)-2-(4-formylphenyl)-4-methyloxazole (3ca)

^{1}H NMR (400 MHz, $CDCl_3$)

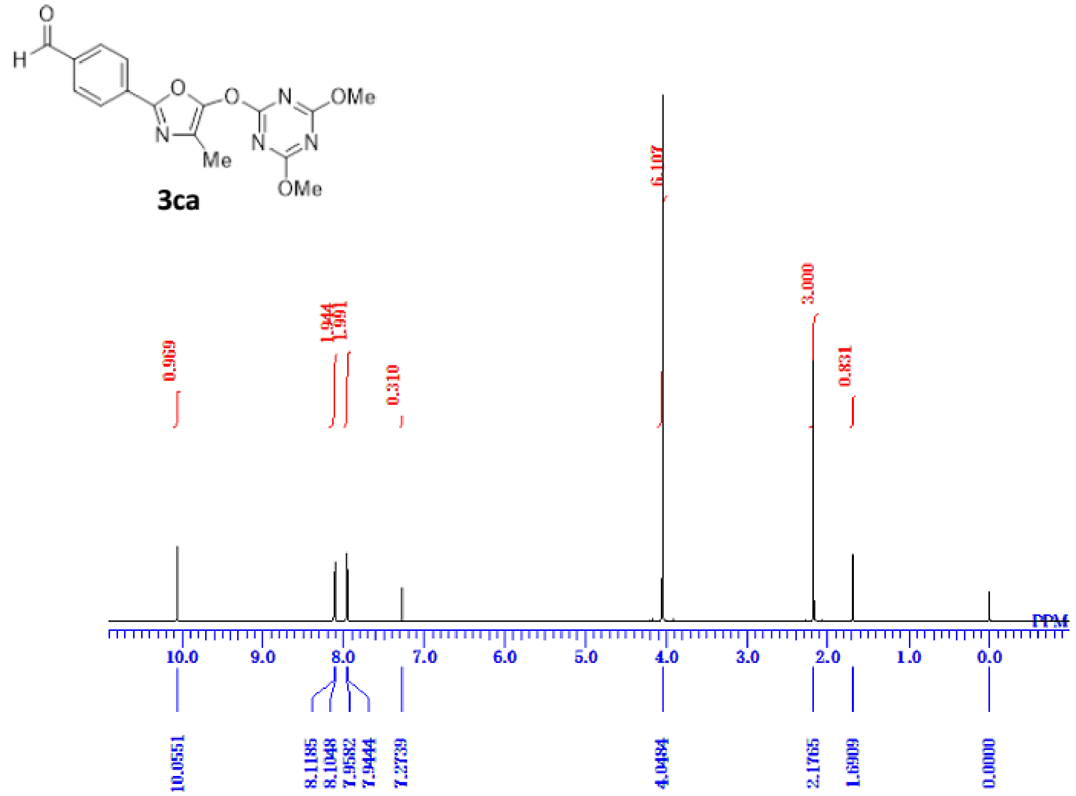

^{13}C NMR (150 MHz, $CDCl_3$)

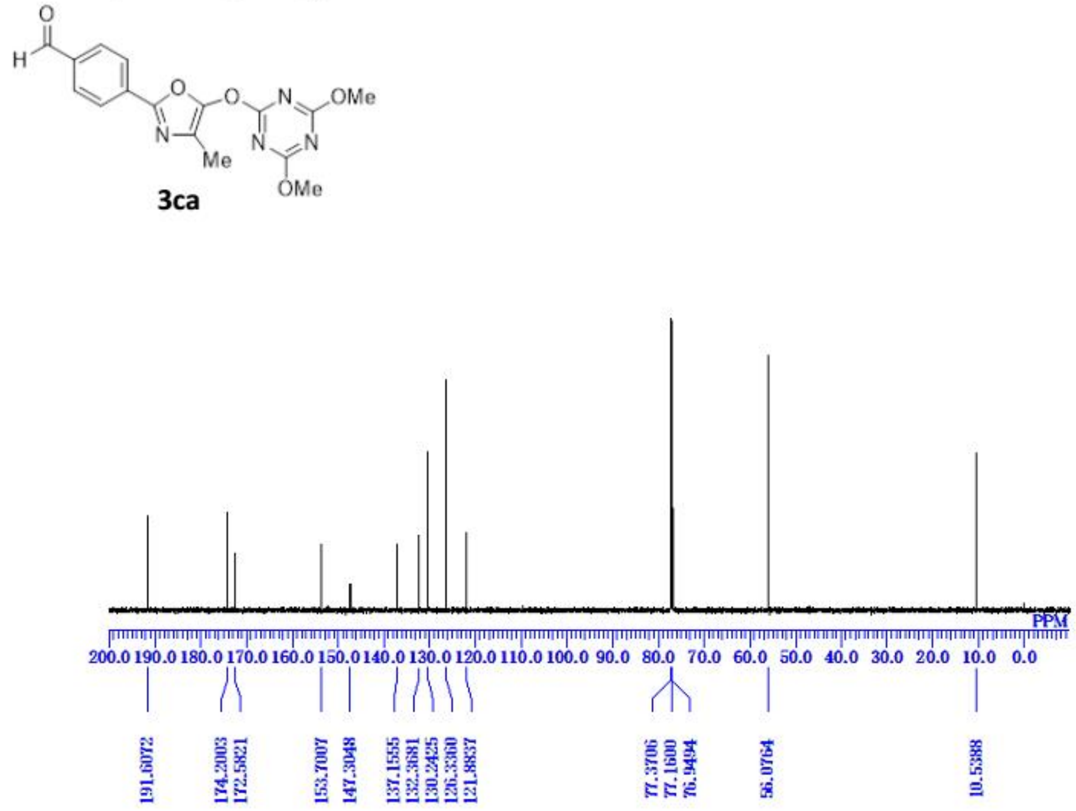

5-((4,6-Dimethoxy-1,3,5-triazin-2-yl)oxy)-4-methyl-2-phenethyloxazole (3da)

^{1}H NMR (600 MHz, $CDCl_3$)

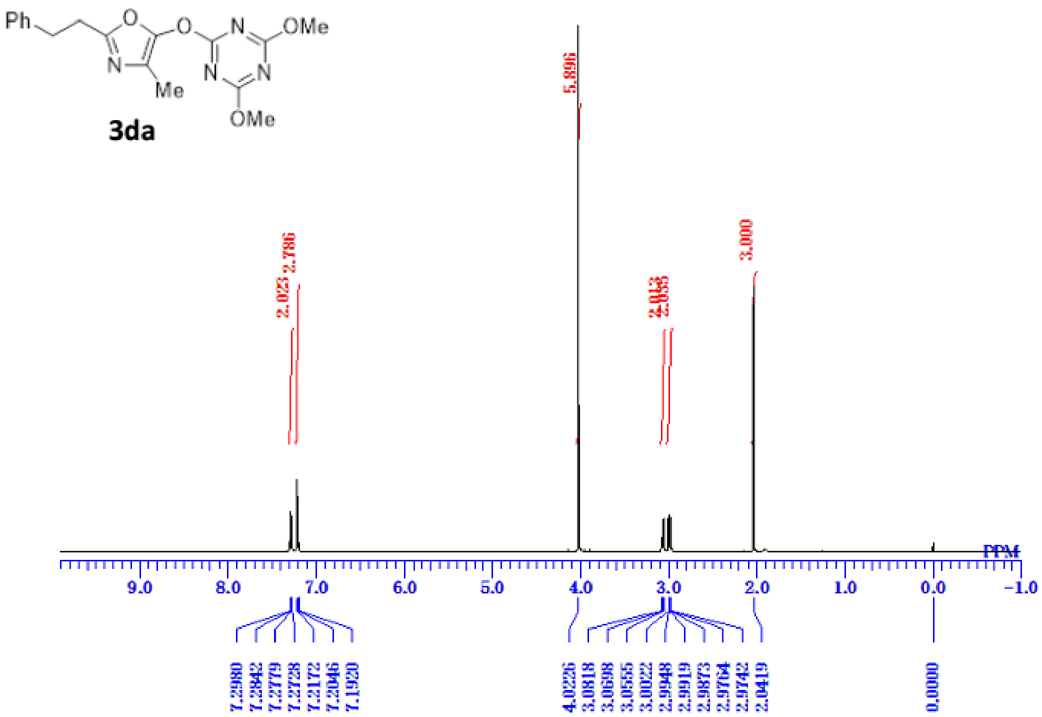

^{13}C NMR (150 MHz, $CDCl_3$)

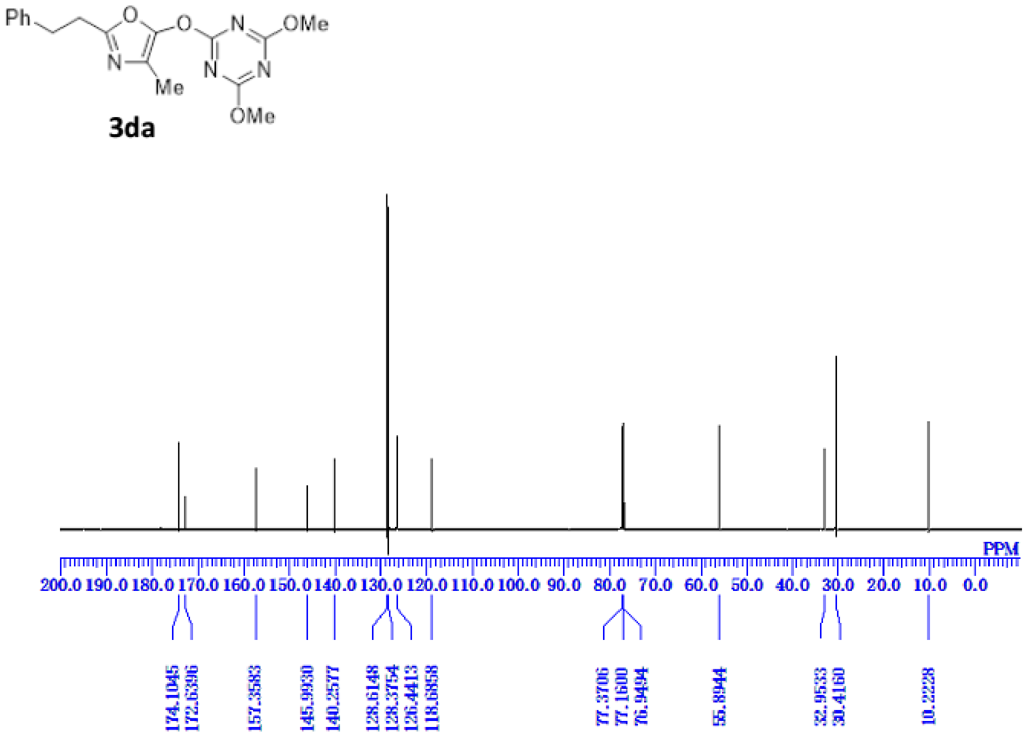

2-(*tert*-Butyl)-5-((4,6-dimethoxy-1,3,5-triazin-2-yl)oxy)-4-methyloxazole (3ea)

^{1}H NMR (400 MHz, $CDCl_3$)

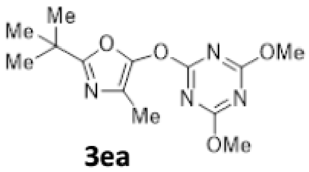

3ea

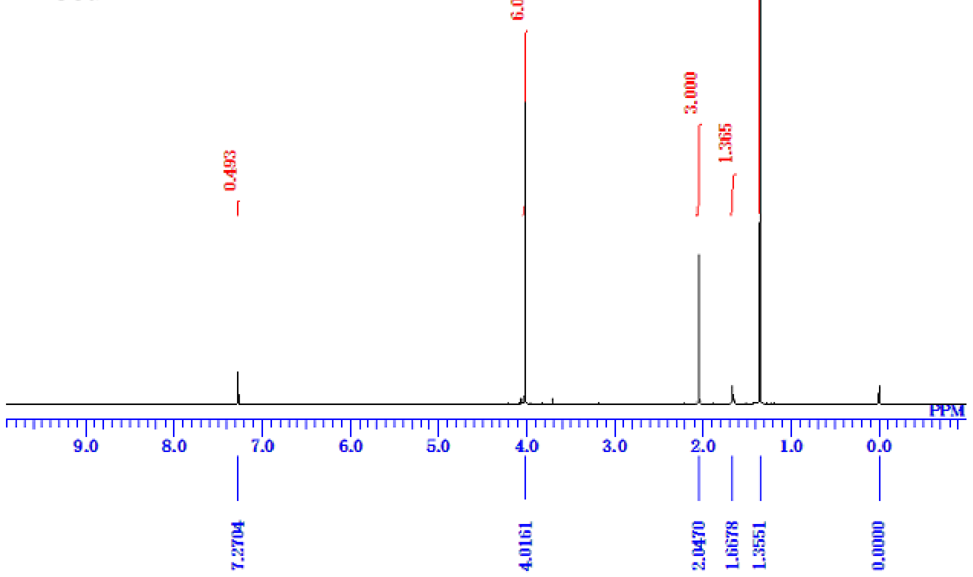

^{13}C NMR (100 MHz, $CDCl_3$)

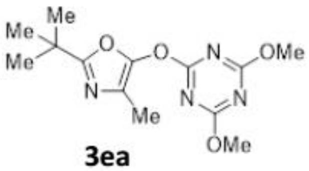

3ea

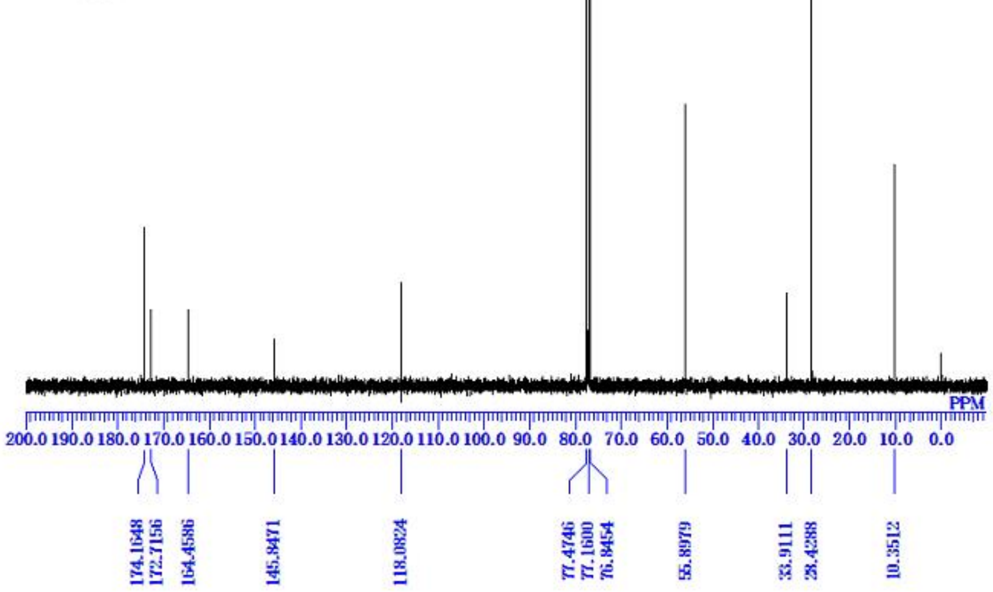

1,4-Bis(5-((4,6-dimethoxy-1,3,5-triazin-2-yl)oxy)-4-methyloxazol-2-yl)benzene (3fa)

^{1}H NMR (400 MHz, $CDCl_3$)

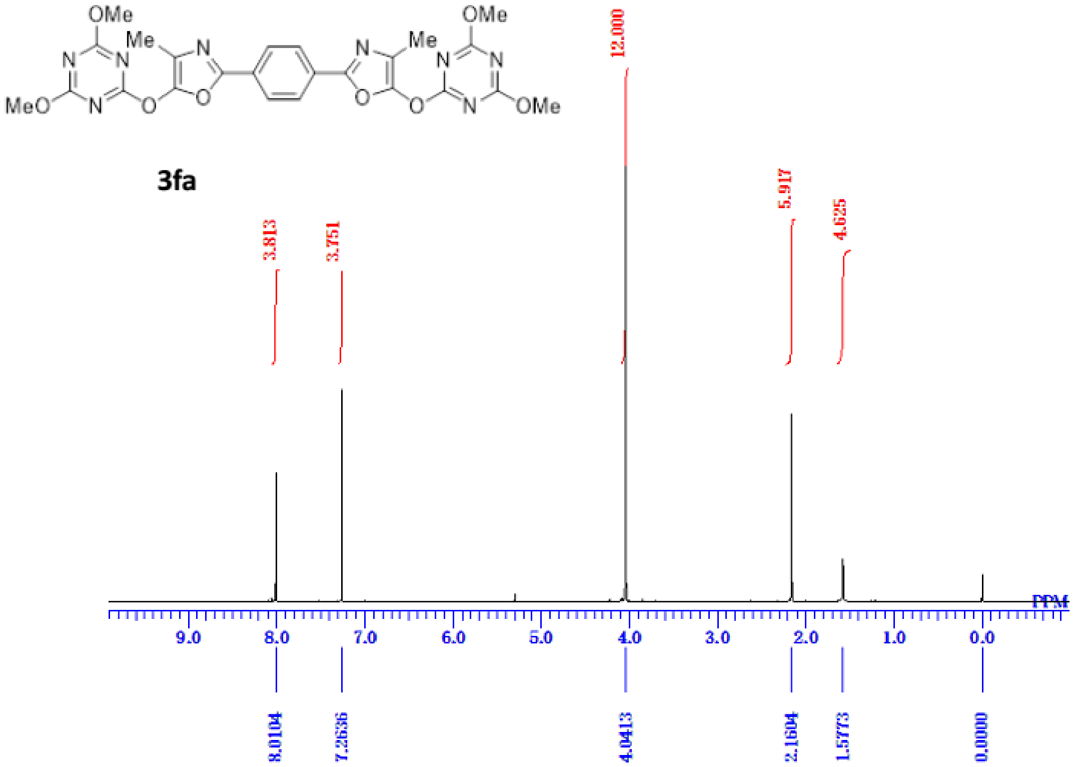

^{13}C NMR (100 MHz, $CDCl_3$)

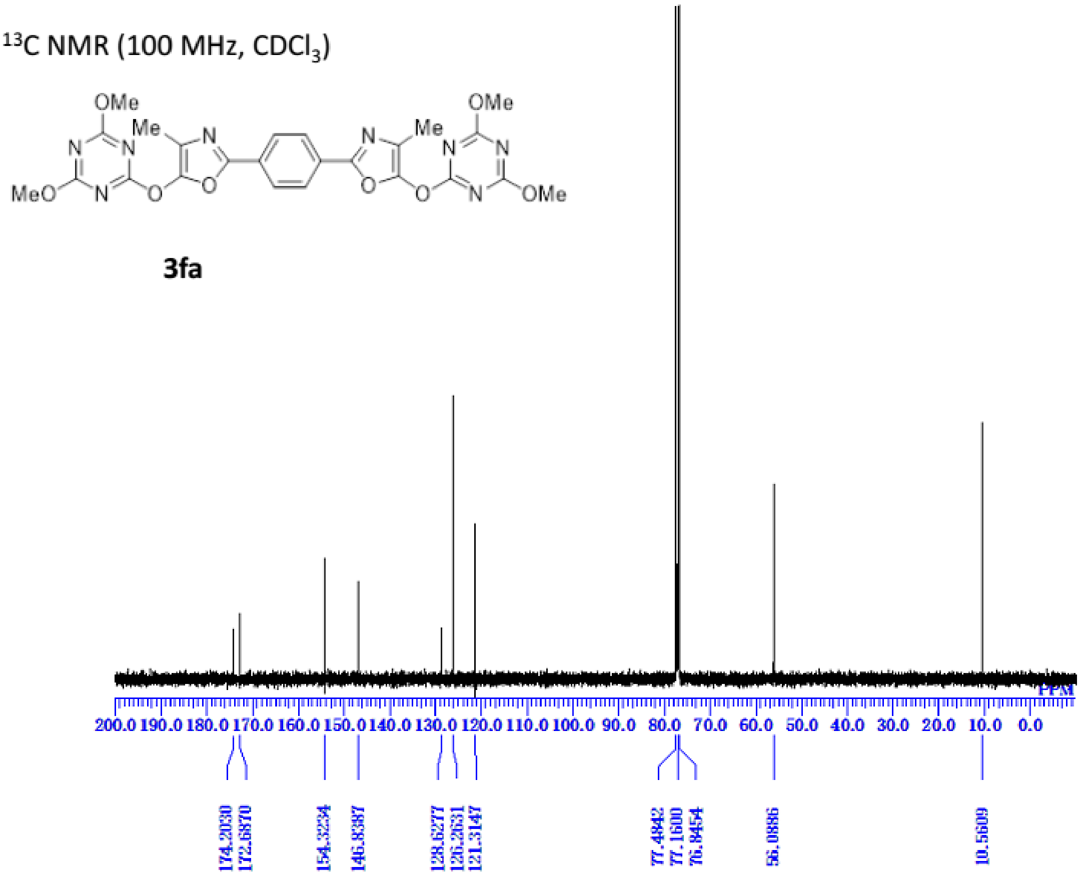

4-Benzyl-5-((4,6-dimethoxy-1,3,5-triazin-2-yl)oxy)-2-phenyloxazole (3ab)

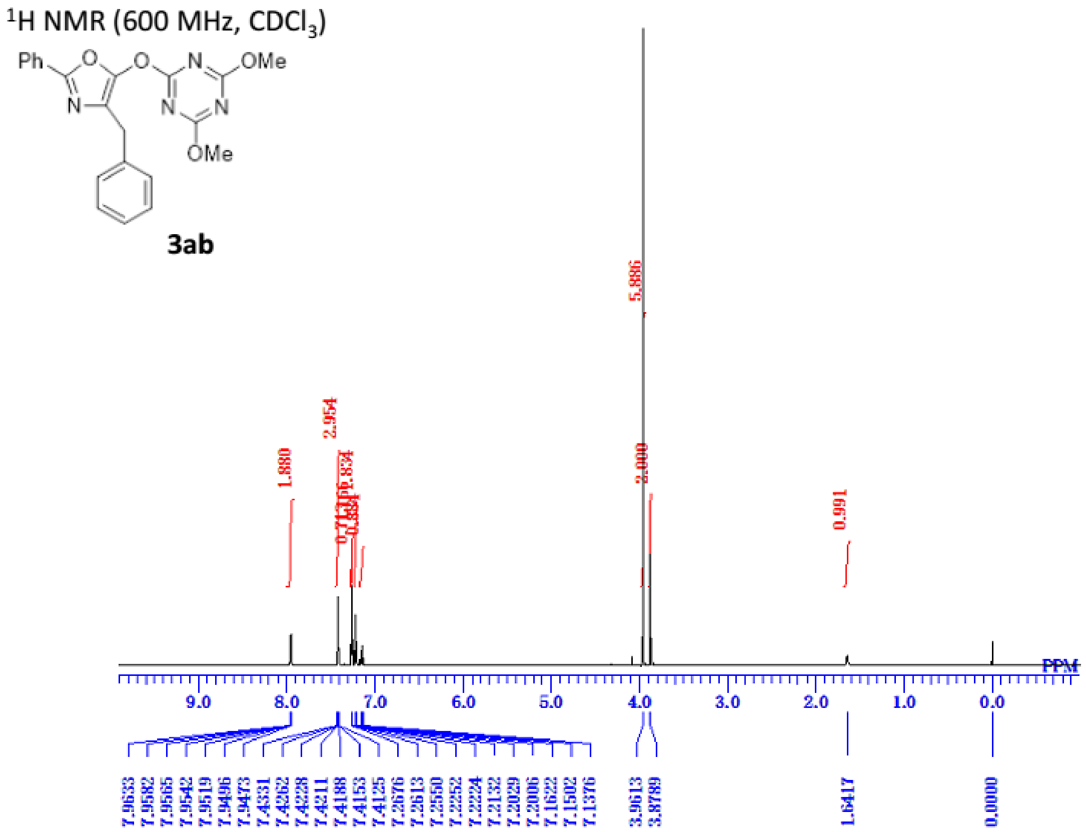

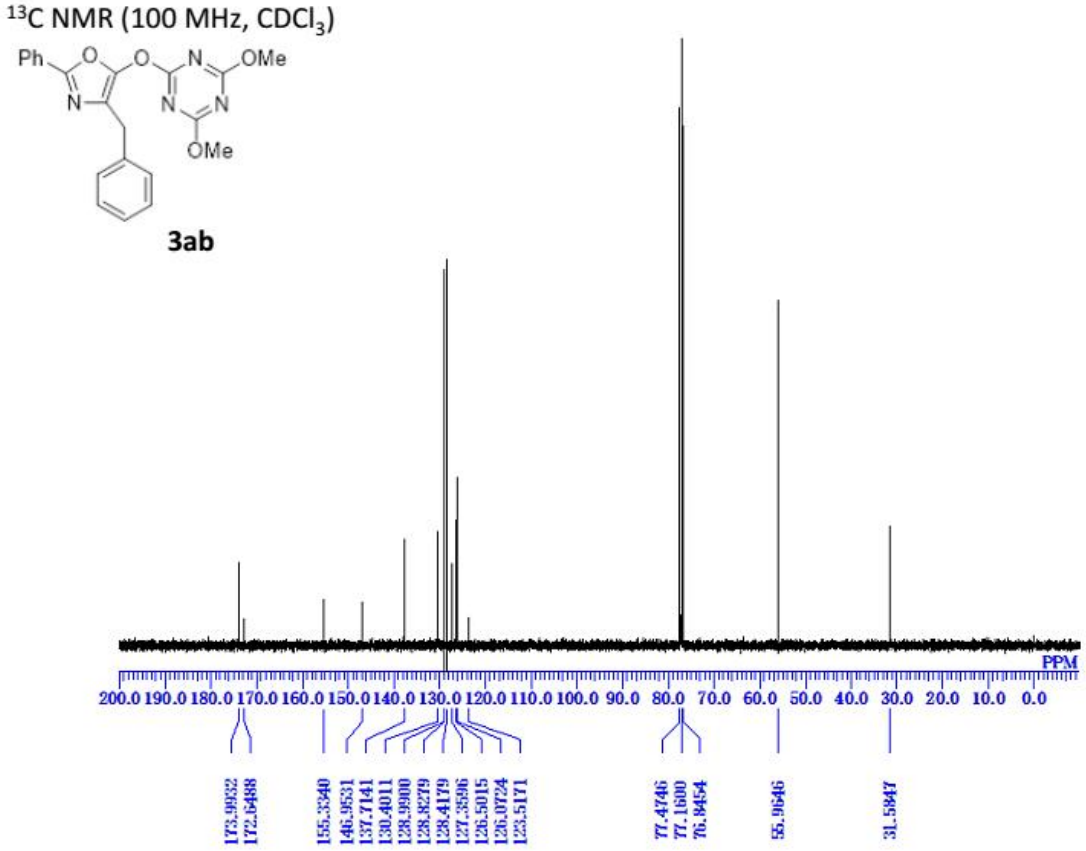

5-((4,6-Dimethoxy-1,3,5-triazin-2-yl)oxy)-4-isopropyl-2-phenyloxazole (3ac)

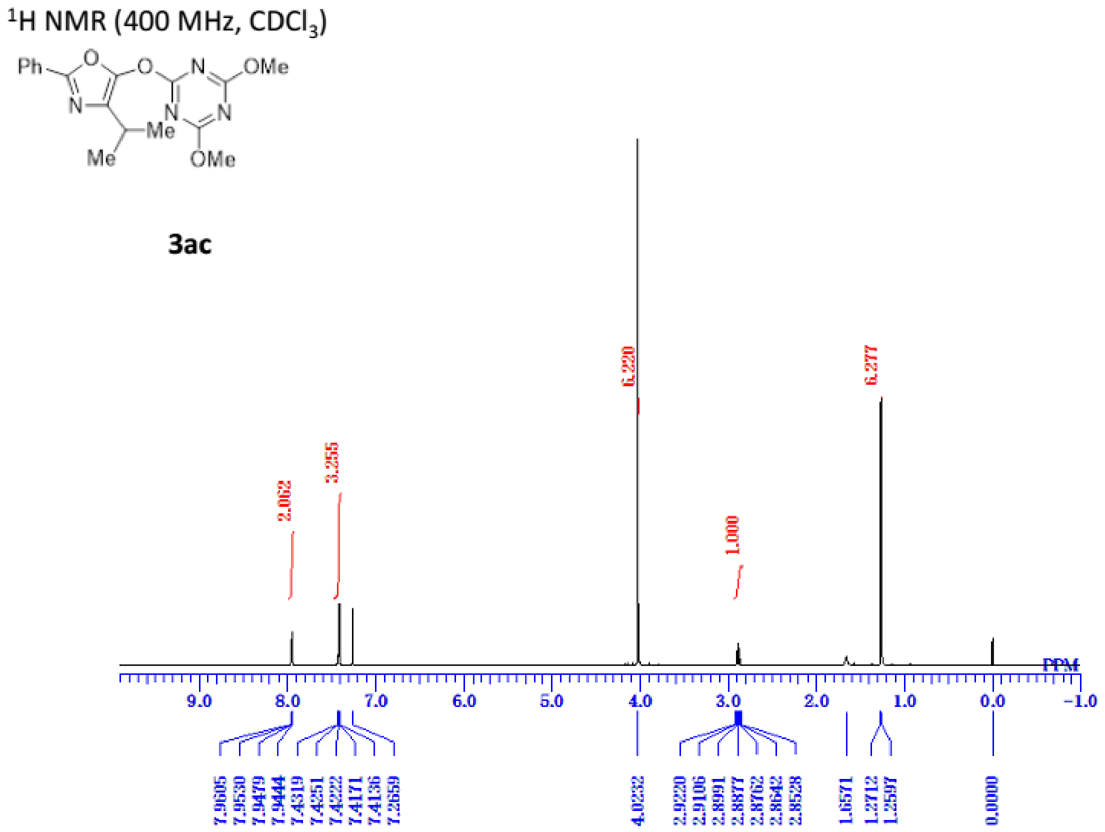

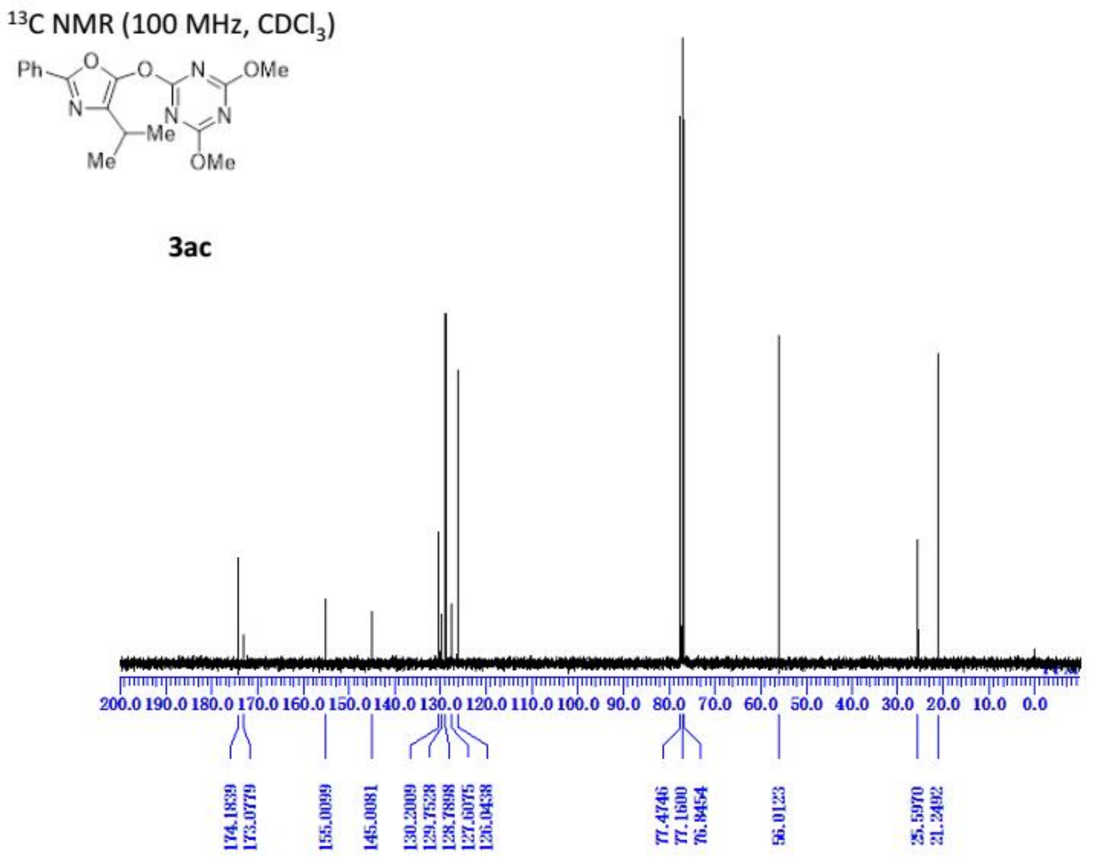

5-((4,6-Dimethoxy-1,3,5-triazin-2-yl)oxy)-4-isobutyl-2-phenyloxazole (3ad)

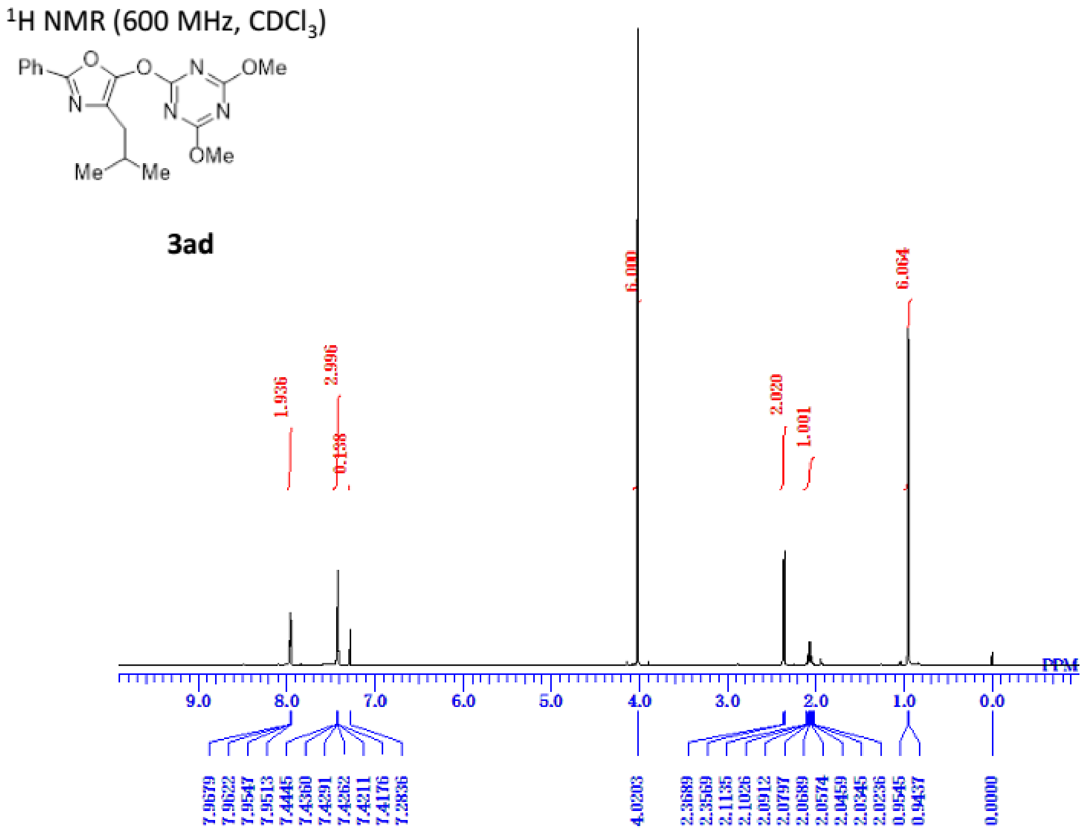

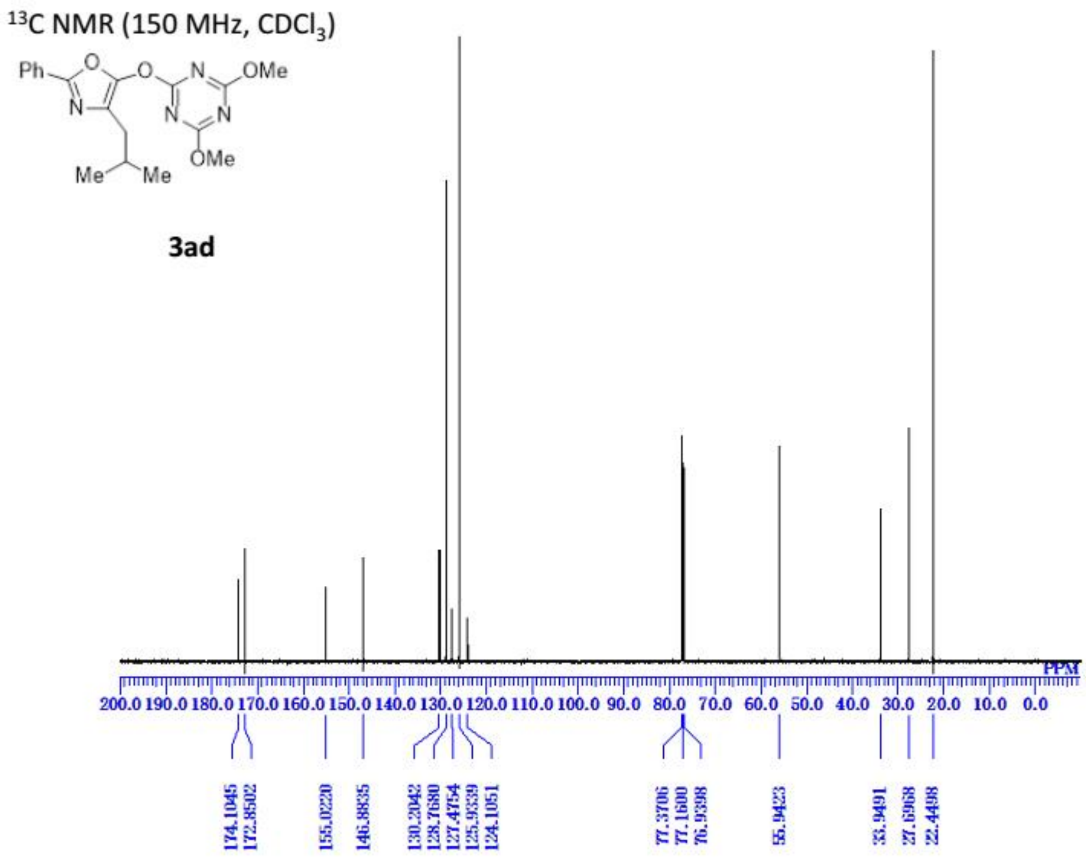

5-((4,6-Dimethoxy-1,3,5-triazin-2-yl)oxy)-4-(2-(methylthio)ethyl)-2-phenyloxazole (3ae)

^1H NMR (600 MHz, $CDCl_3$)

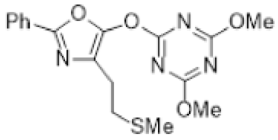

3ae

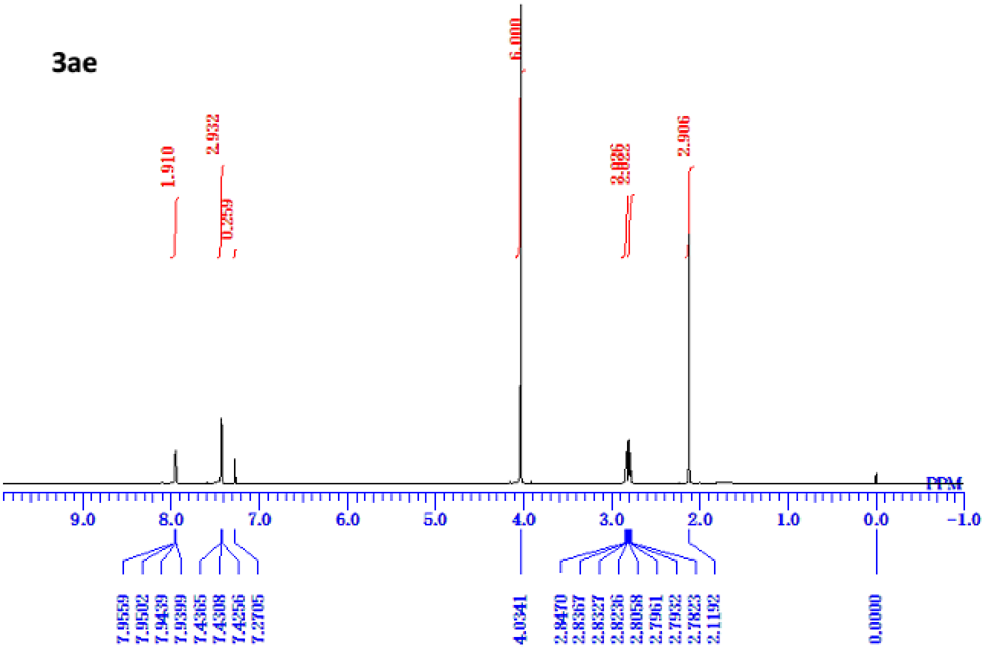

^{13}C NMR (150 MHz, $CDCl_3$)

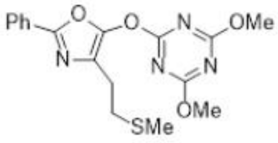

3ae

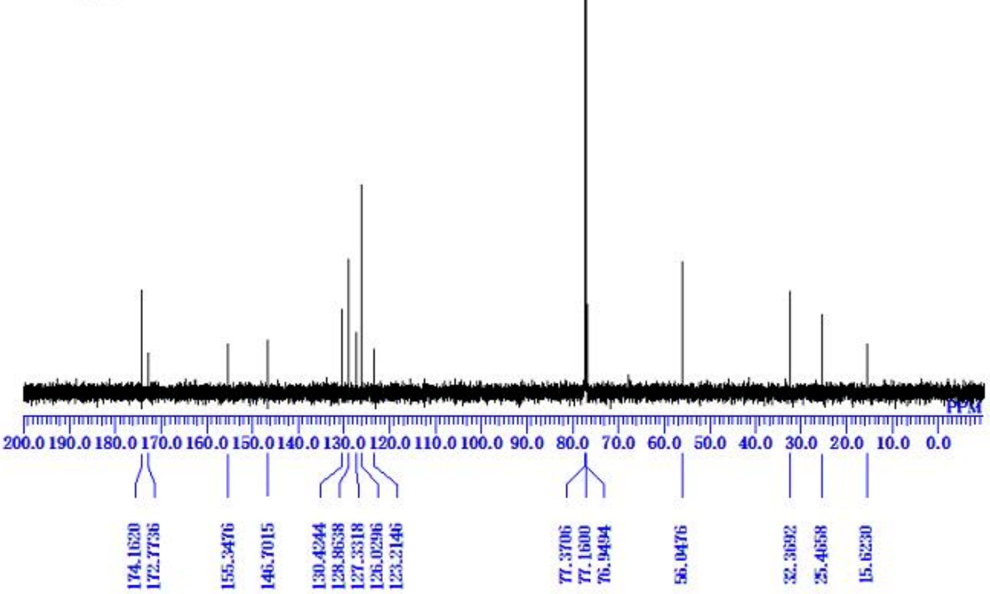

5-((4,6-Dimethoxy-1,3,5-triazin-2-yl)oxy)-2,4-diphenyloxazole (3af)

^{1}H NMR (600 MHz, $CDCl_3$)

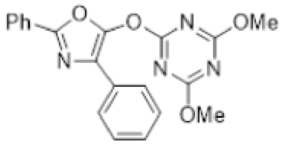

3af

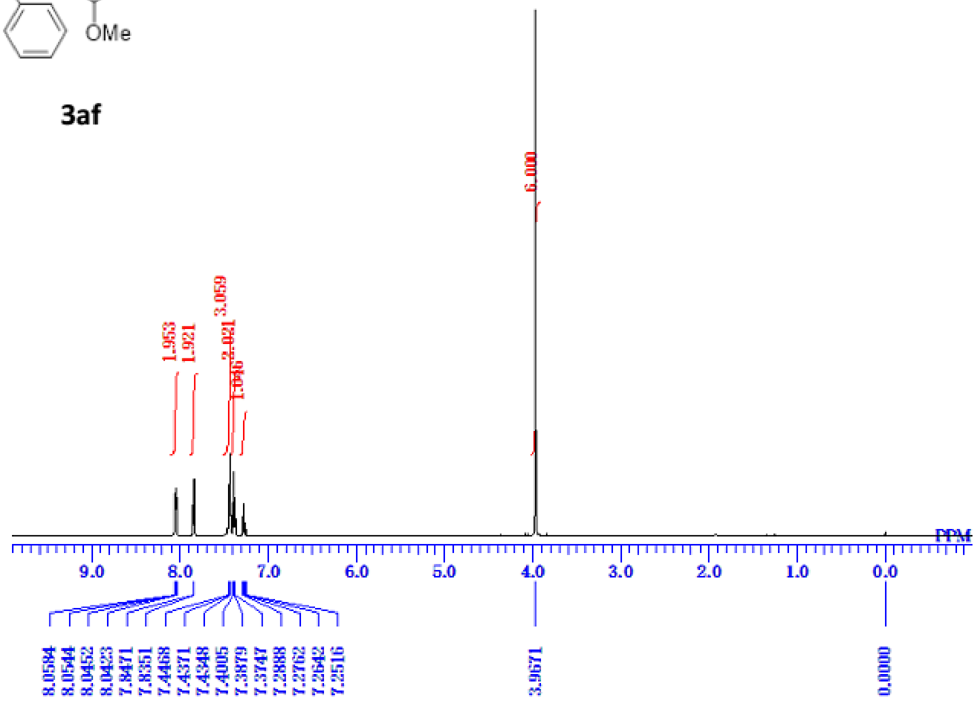

^{13}C NMR (150 MHz, $CDCl_3$)

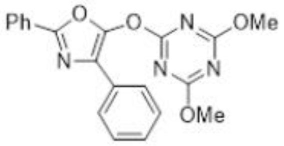

3af

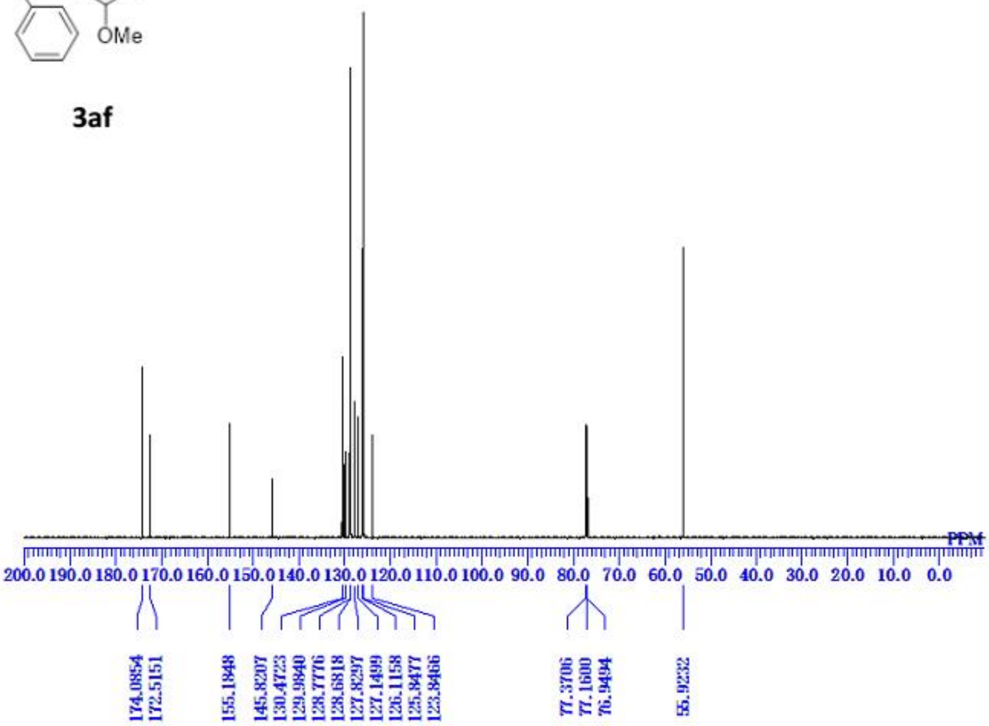

4-Methyl-2,5-diphenyloxazole (5aaa)

^{1}H NMR (400 MHz, $CDCl_3$)

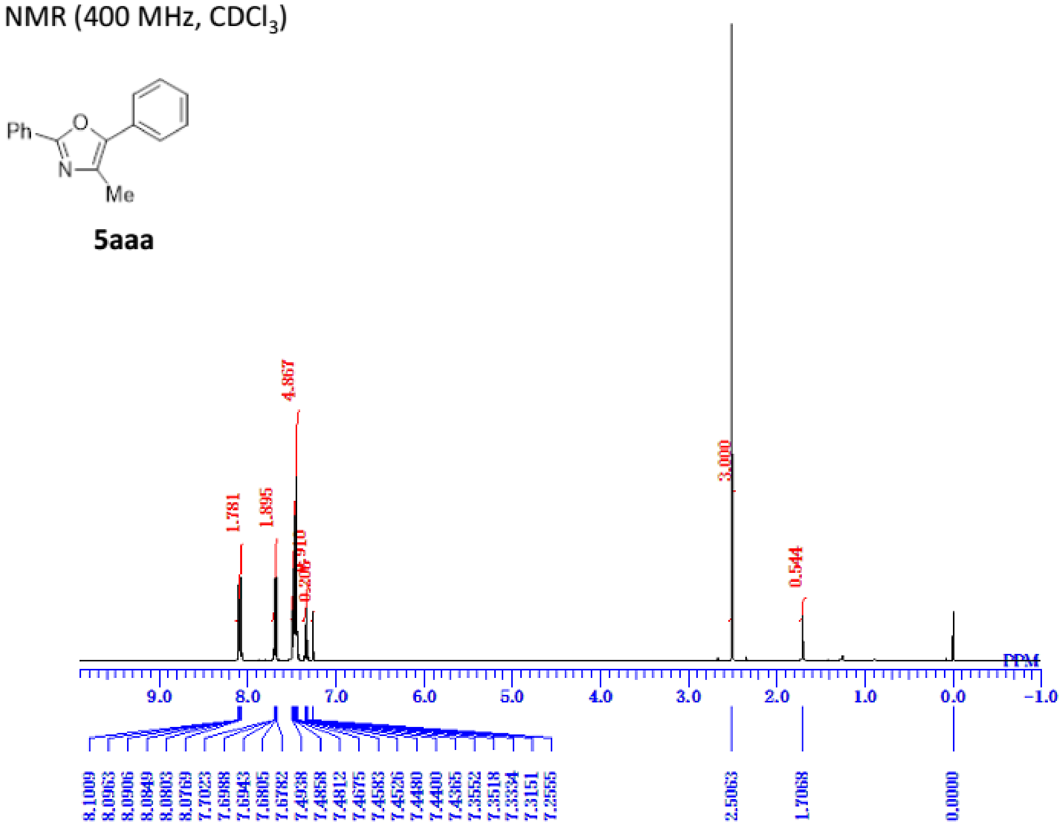

^{13}C NMR (100 MHz, $CDCl_3$)

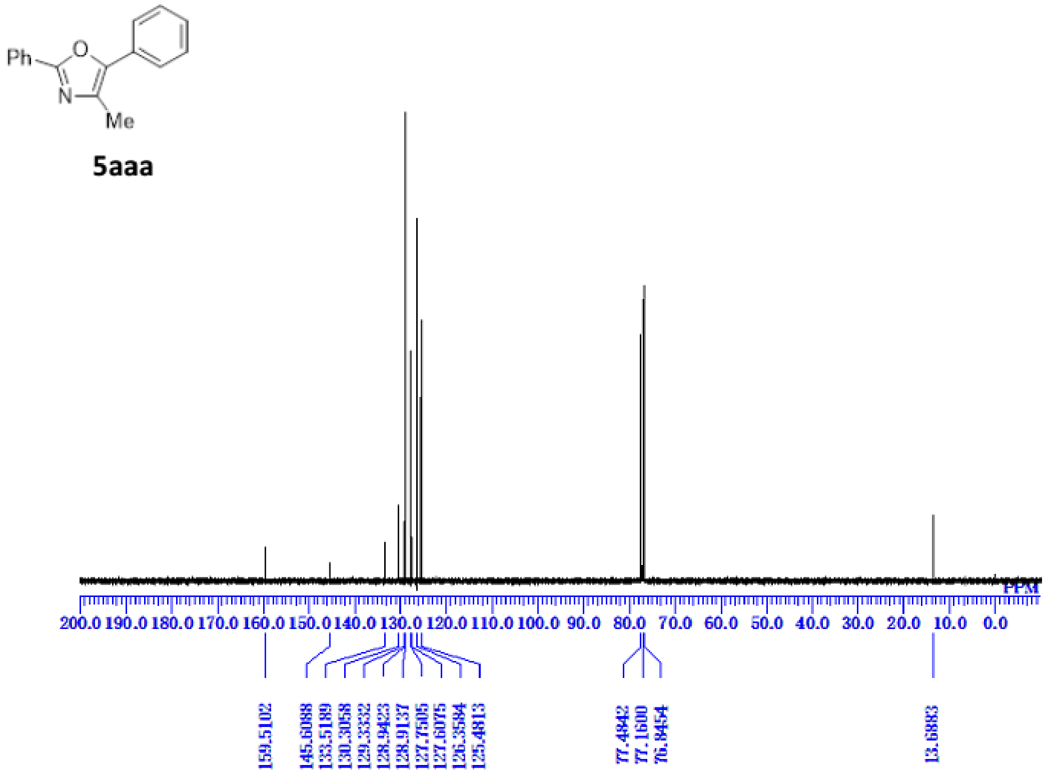

4-Methyl-2-phenyl-5-(4-ethoxycarbonylphenyl)oxazole (5aab)

^{1}H NMR (600 MHz, $CDCl_3$)

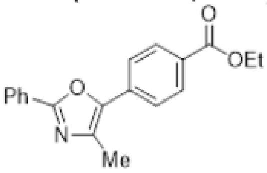

5aab

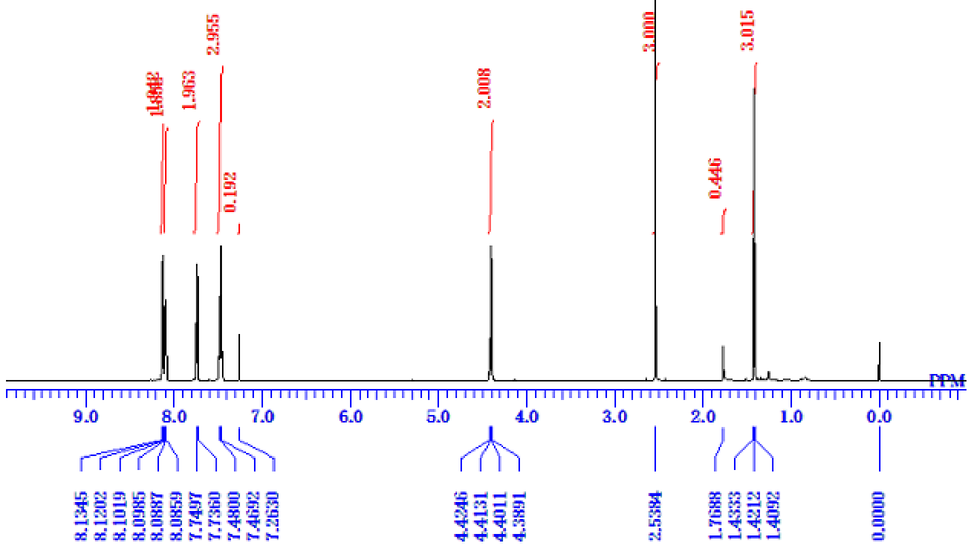

^{13}C NMR (150 MHz, $CDCl_3$)

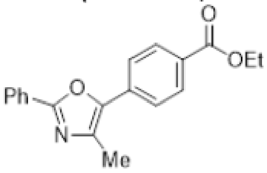

5aab

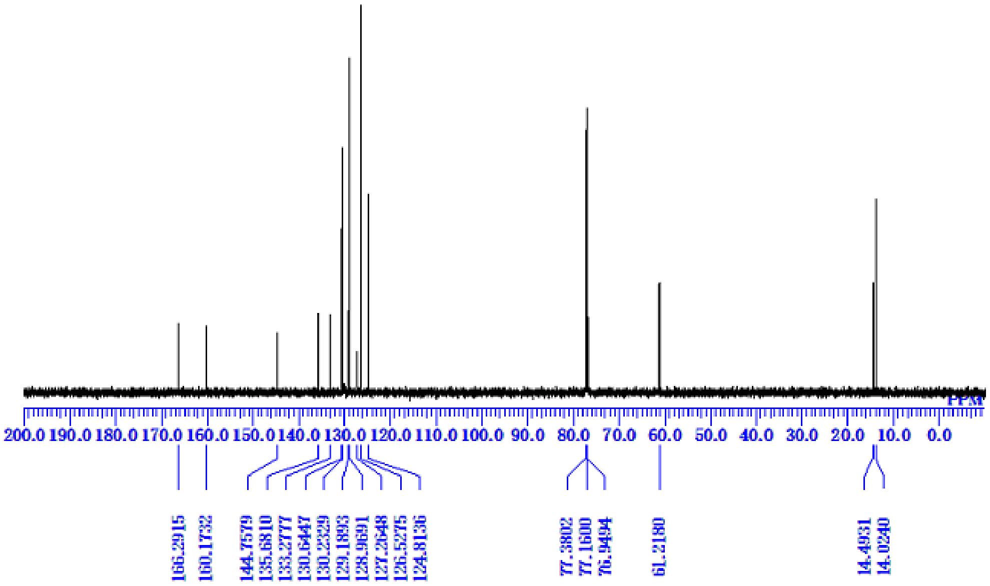

4-Methyl-5-(4-methoxyphenyl)-2-phenyloxazole (5aac)

^{1}H NMR (600 MHz, $CDCl_3$)

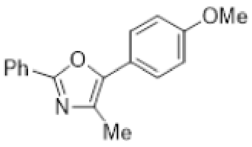

5aac

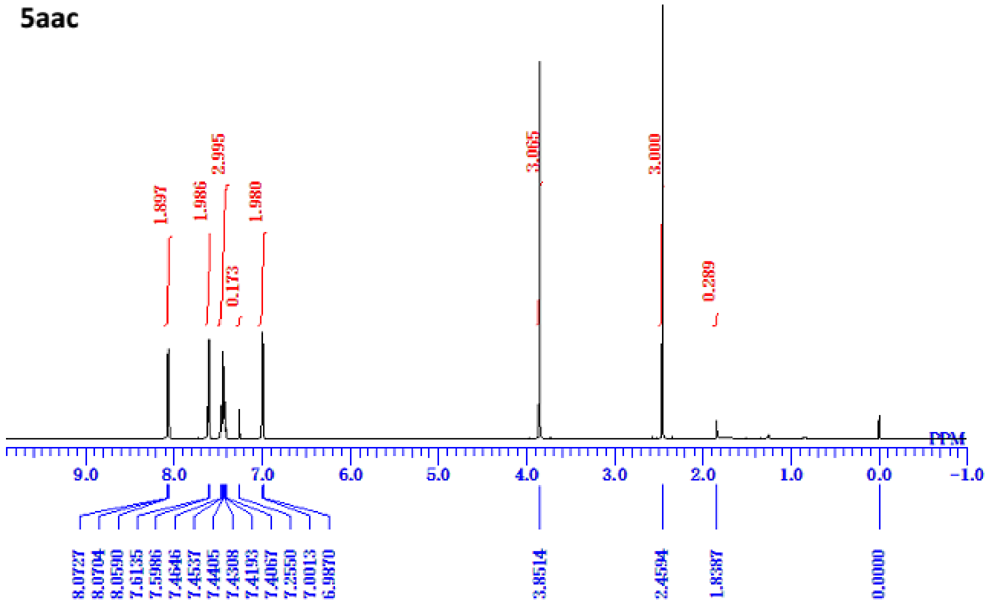

^{13}C NMR (150 MHz, $CDCl_3$)

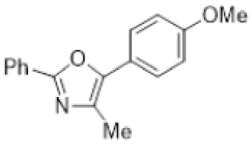

5aac

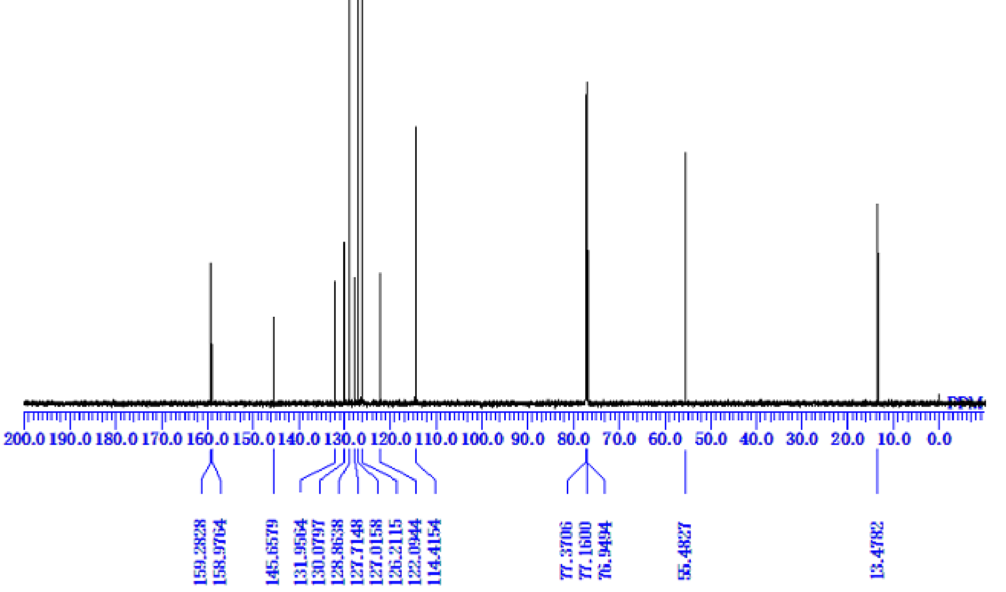

4-Methyl-5-(2-naphtyl)-2-phenyloxazole (5aad)

^{1}H NMR (600 MHz, $CDCl_3$)

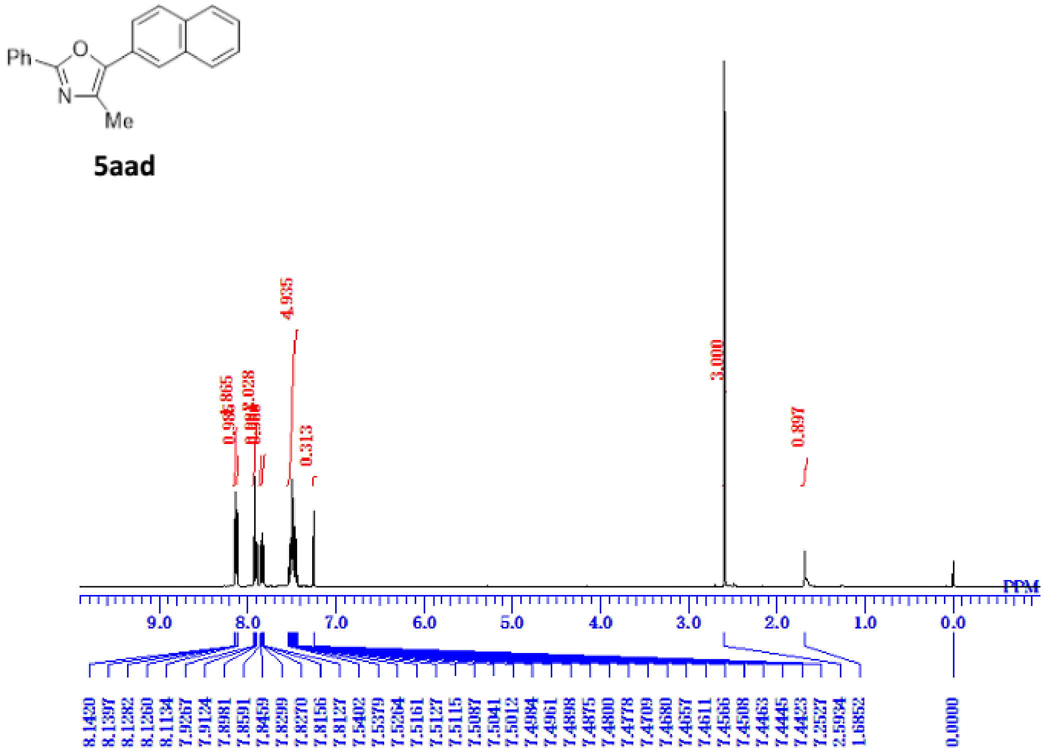

^{13}C NMR (150 MHz, $CDCl_3$)

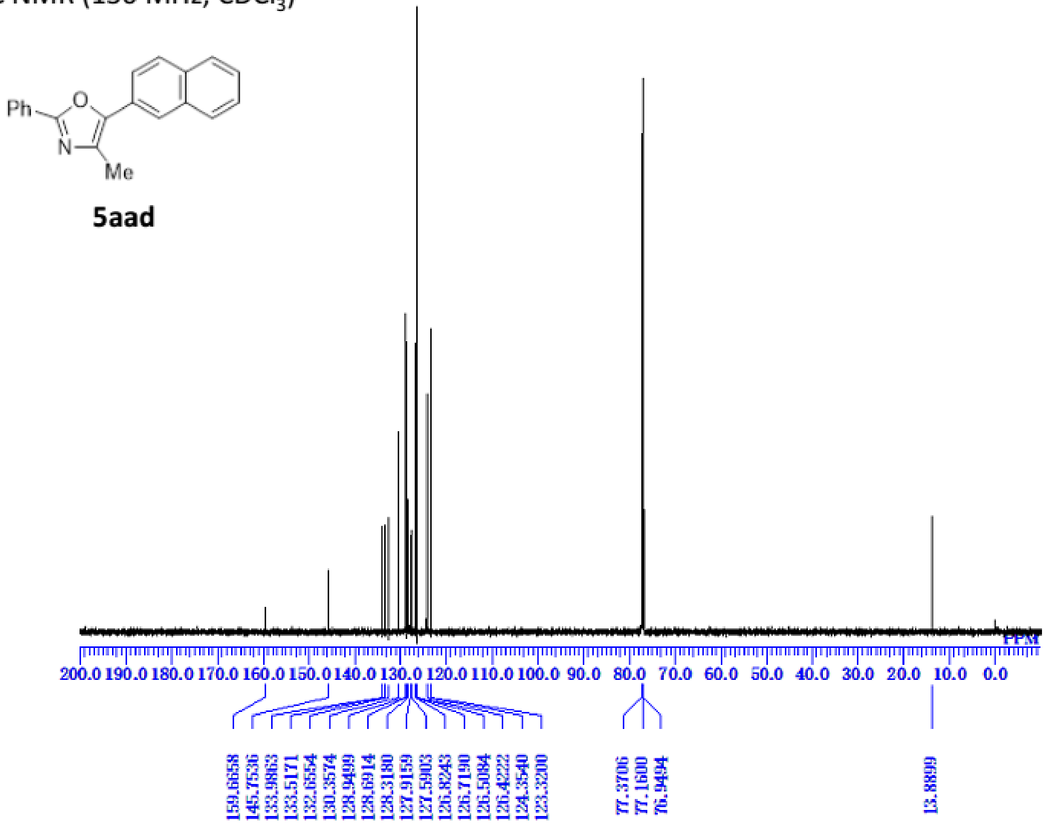

4-Methyl-2-phenyl-5-(4-tolyl)oxazole (5aae)

^{1}H NMR (600 MHz, $CDCl_3$)

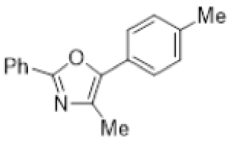

5aae

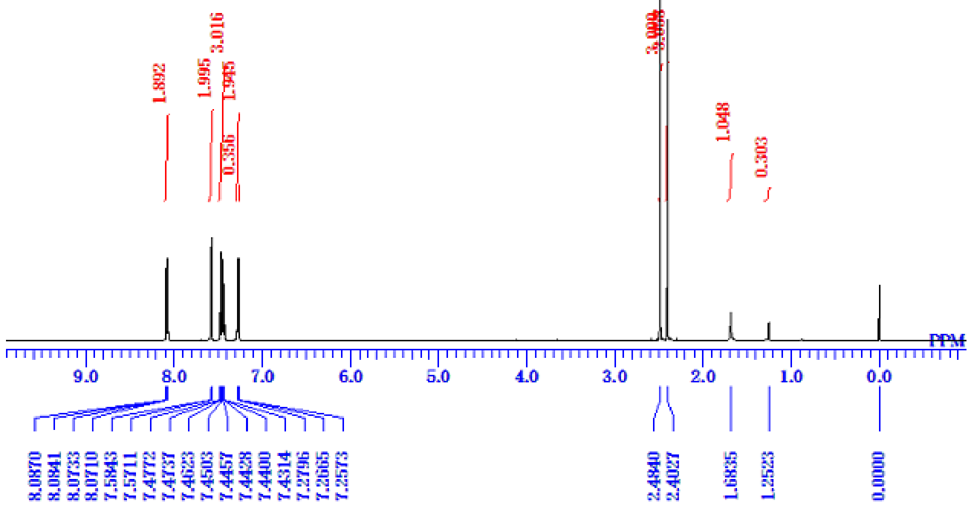

^{13}C NMR (150 MHz, $CDCl_3$)

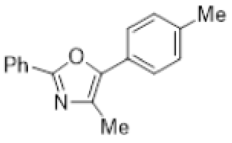

5aae

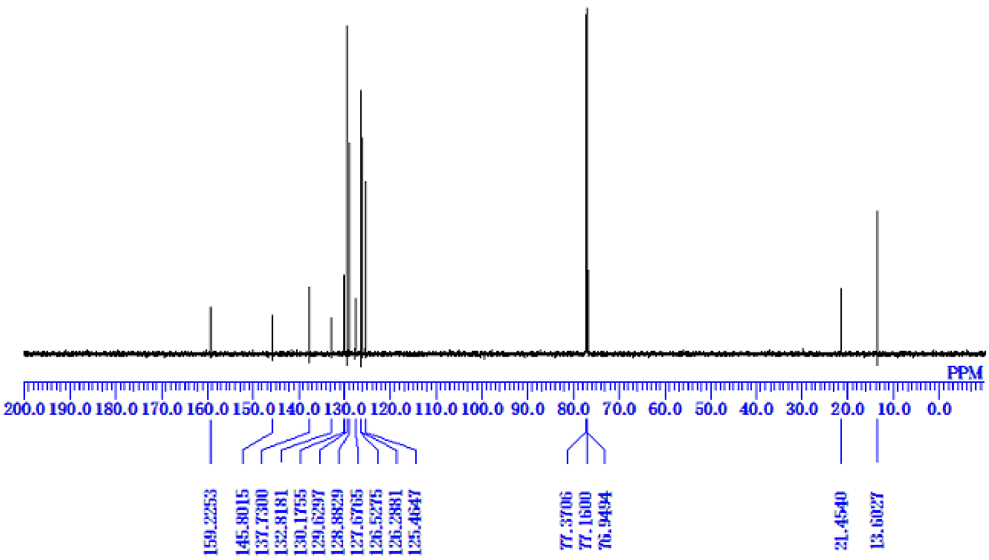

4-Methyl-2-phenyl-5-(2-tolyl)oxazole (5aaf)

^{1}H NMR (600 MHz, $CDCl_3$)

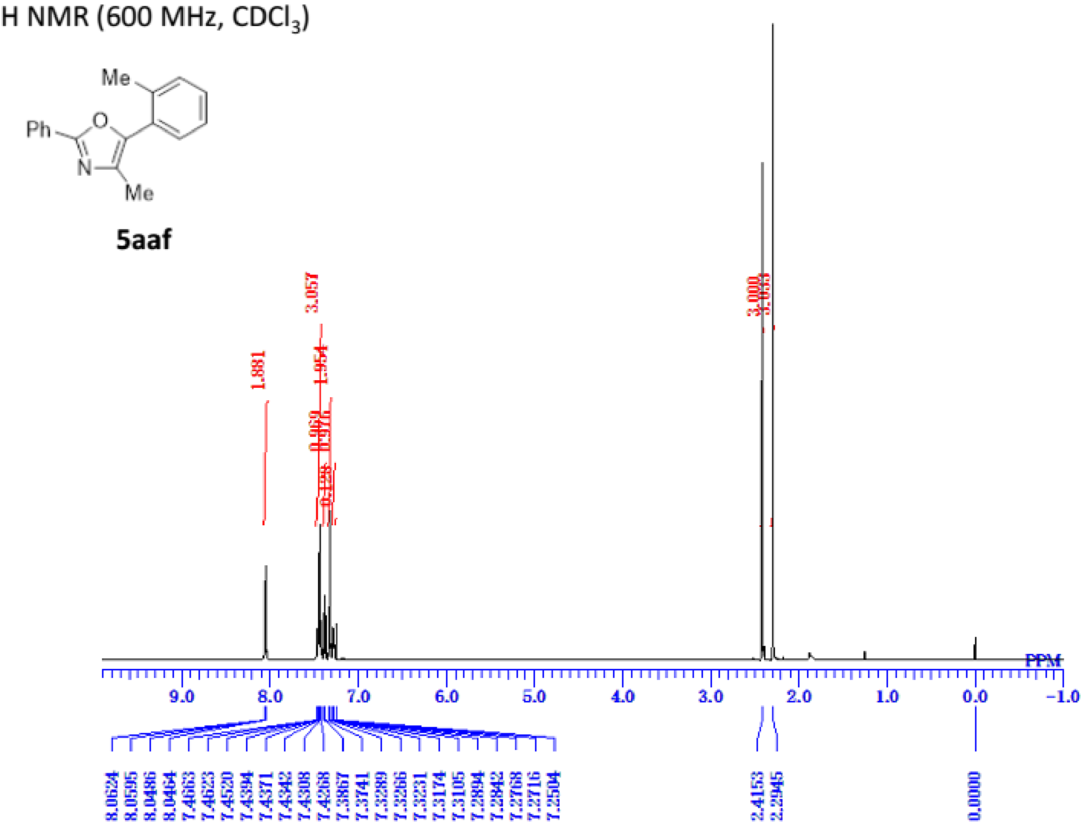

^{13}C NMR (150 MHz, $CDCl_3$)

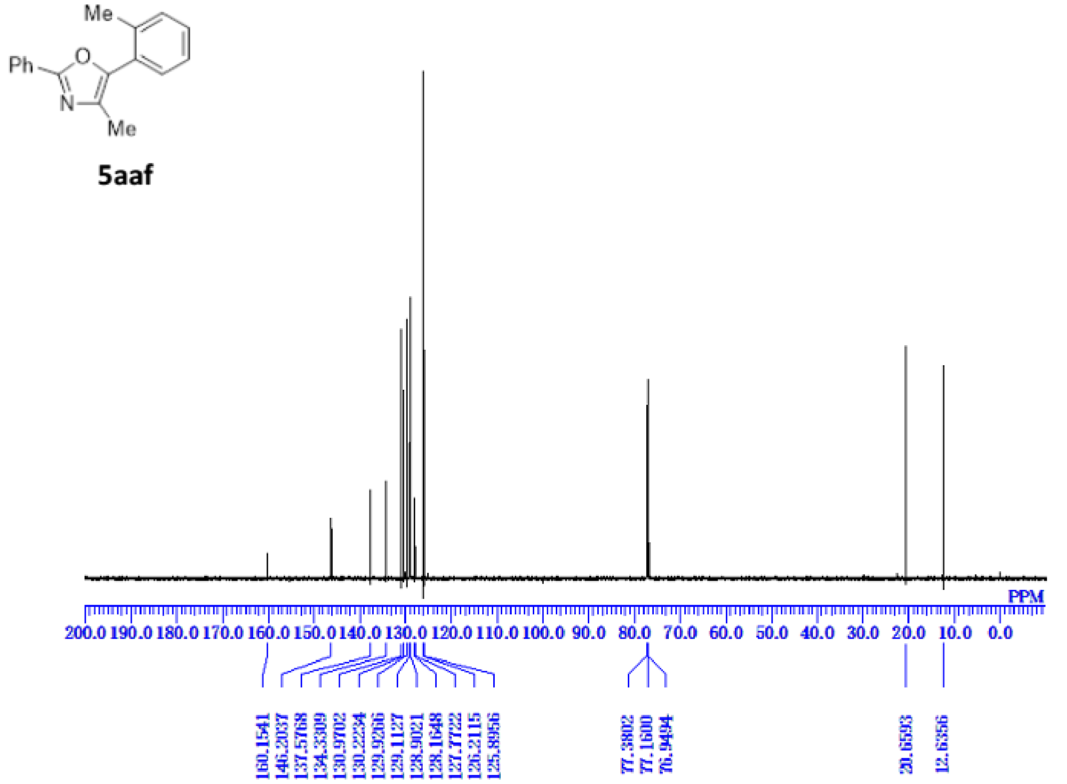

4-Methyl-2-phenyl-5-(3-thienyl)oxazole (5aag)

^{1}H NMR (600 MHz, $CDCl_3$)

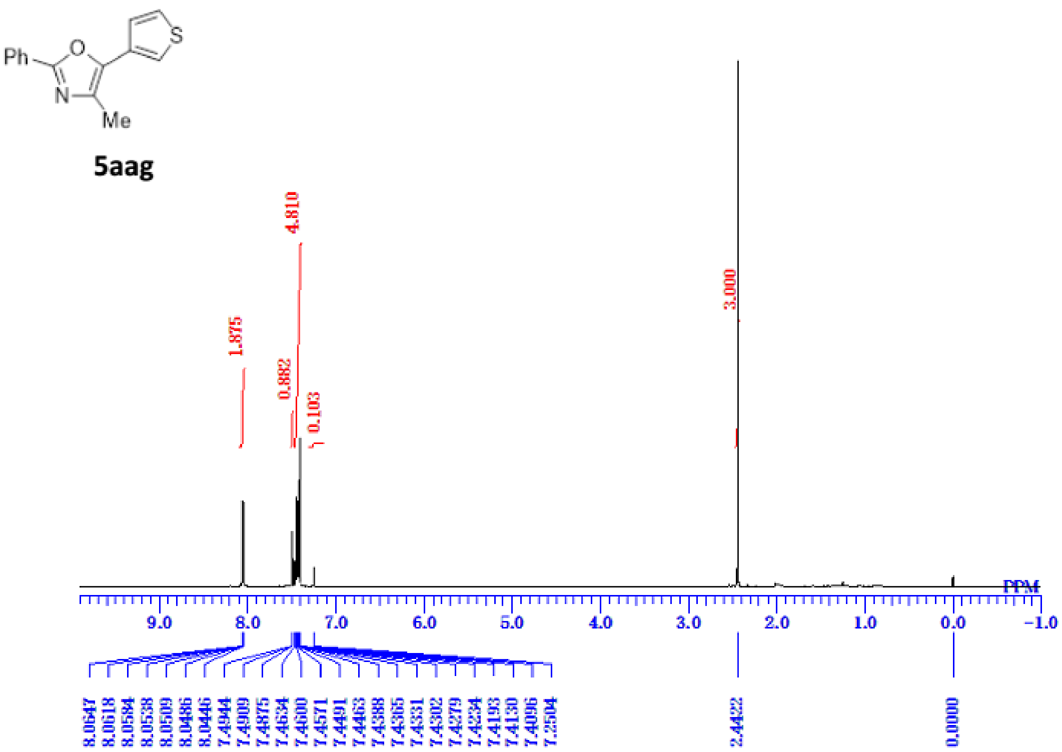

^{13}C NMR (150 MHz, $CDCl_3$)

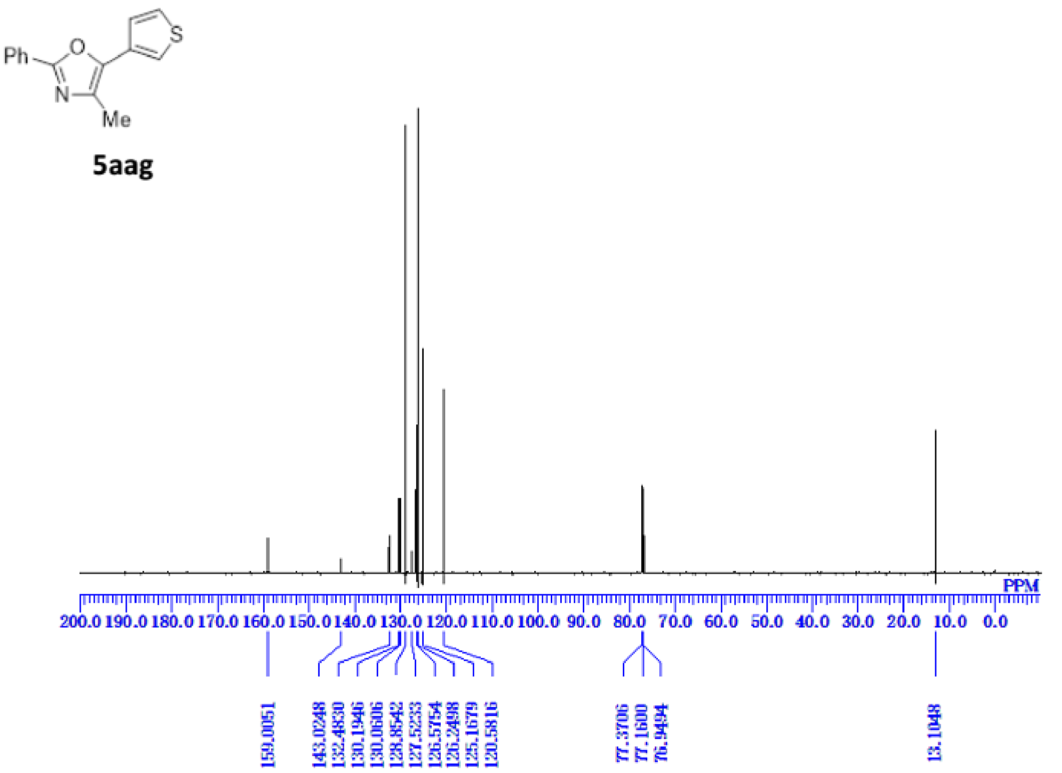

4-Methyl-2-(4-methoxyphenyl)-5-phenyloxazole (5baa)

^{1}H NMR (600 MHz, $CDCl_3$)

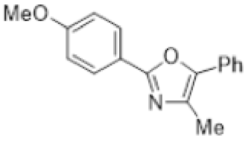

5baa

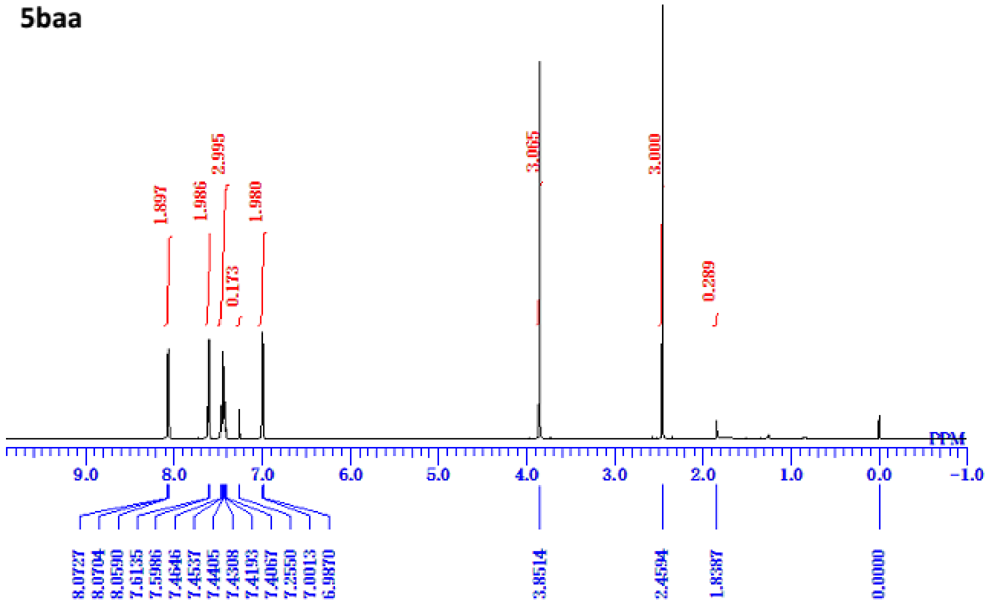

^{13}C NMR (150 MHz, $CDCl_3$)

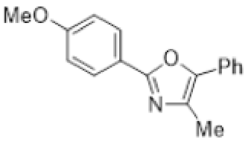

5baa

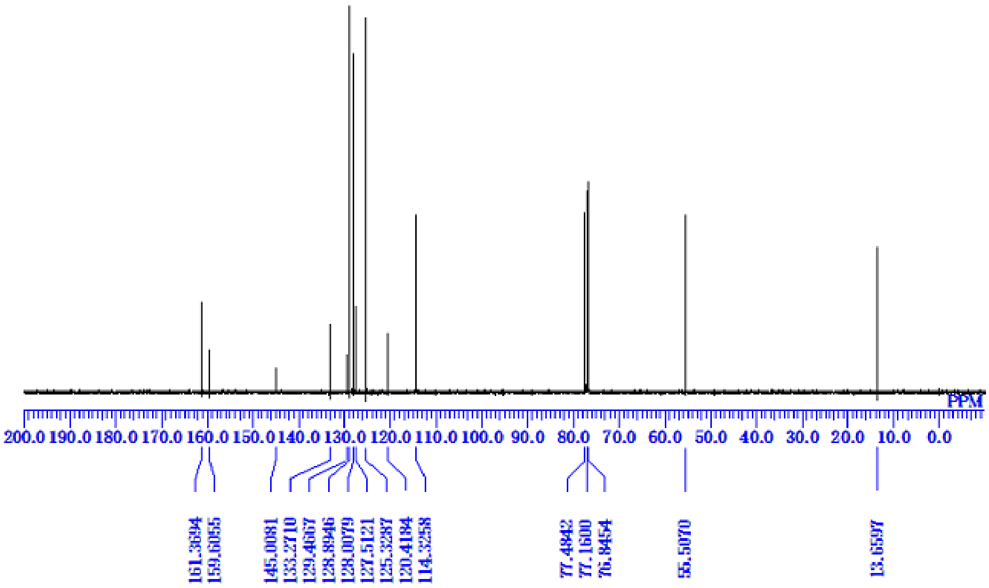

2-(4-Formylphenyl)-4-methyl-5-phenyloxazole (5caa)

^{1}H NMR (600 MHz, $CDCl_3$)

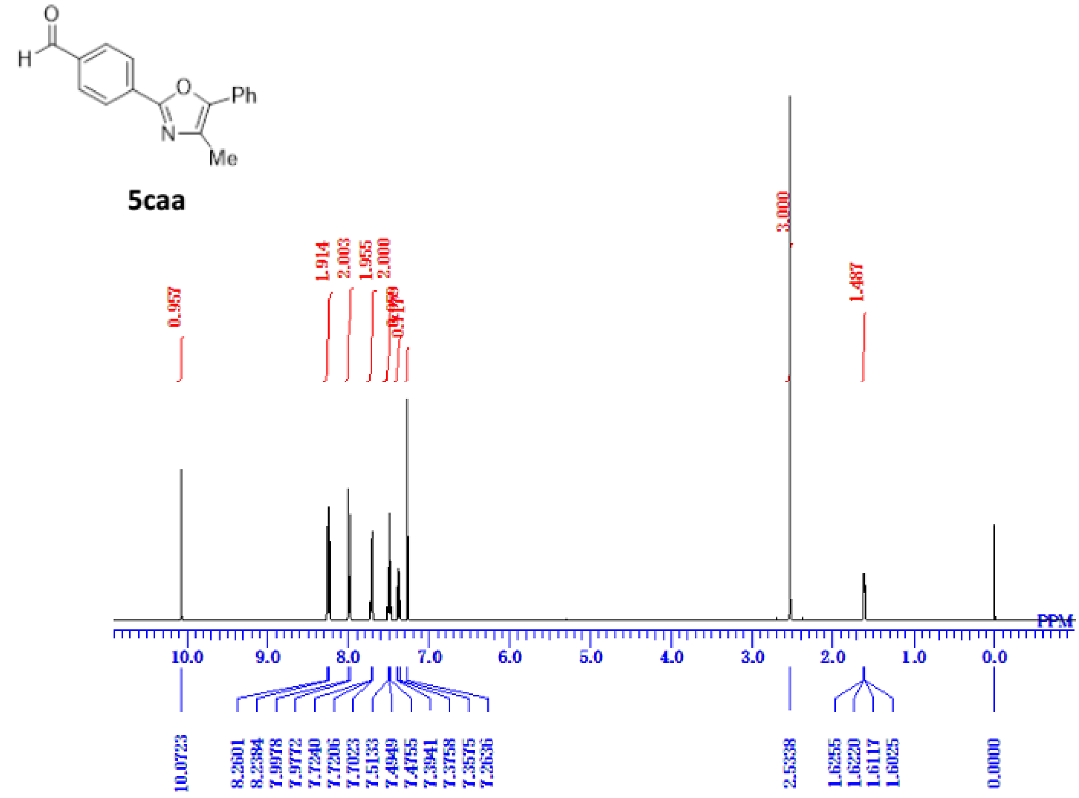

^{13}C NMR (150 MHz, $CDCl_3$)

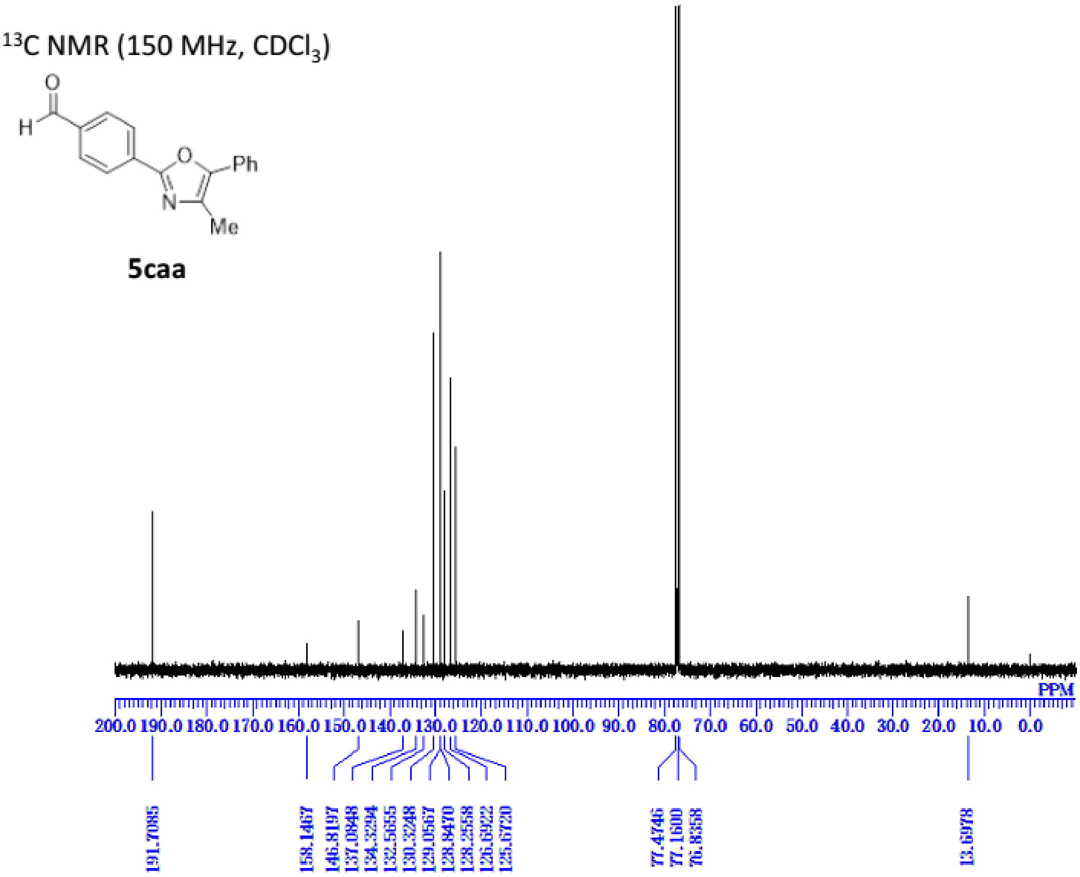

4-Methyl-2-phenethyl-5-phenyloxazole (5daa)

^{1}H NMR (600 MHz, $CDCl_3$)

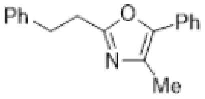

5daa

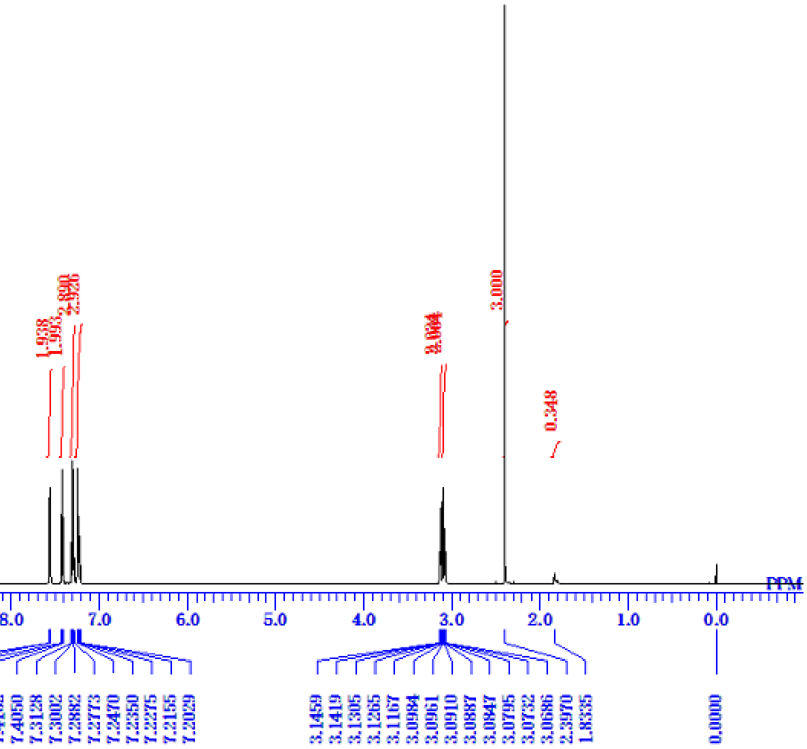

^{13}C NMR (150 MHz, $CDCl_3$)

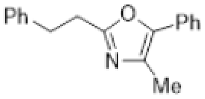

5daa

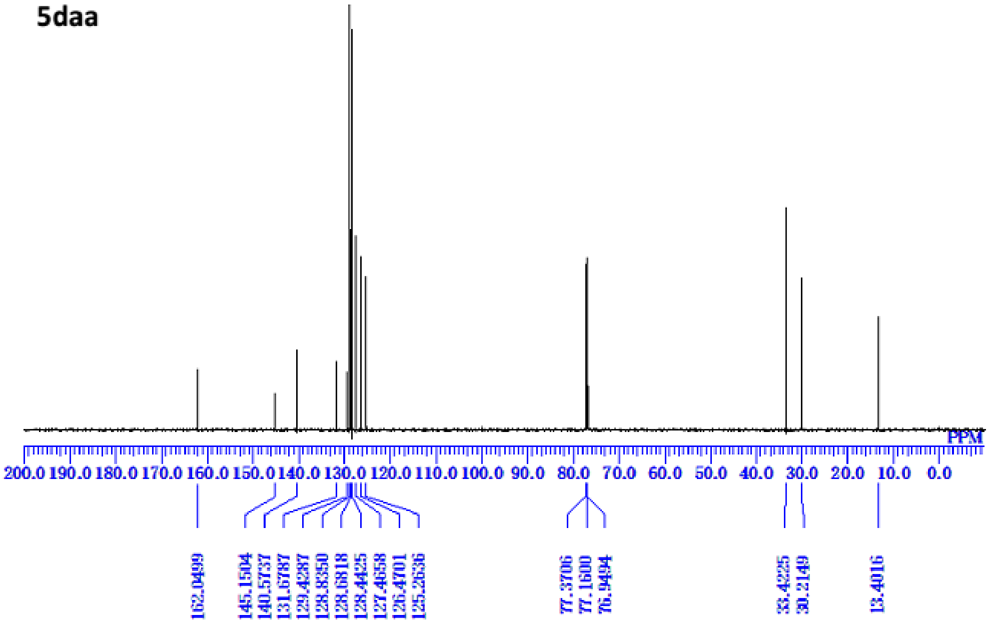

4-Methyl-2-*tert*-butyl-5-phenyloxazole (5eaa)

^{1}H NMR (600 MHz, $CDCl_3$)

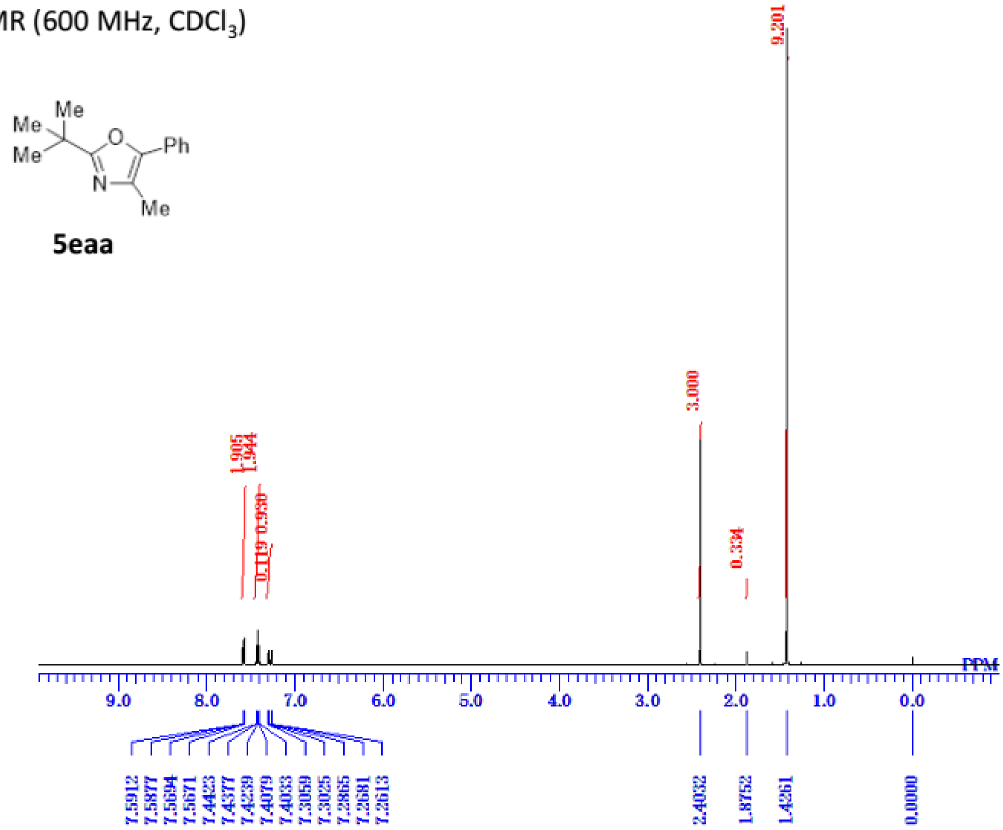

^{13}C NMR (150 MHz, $CDCl_3$)

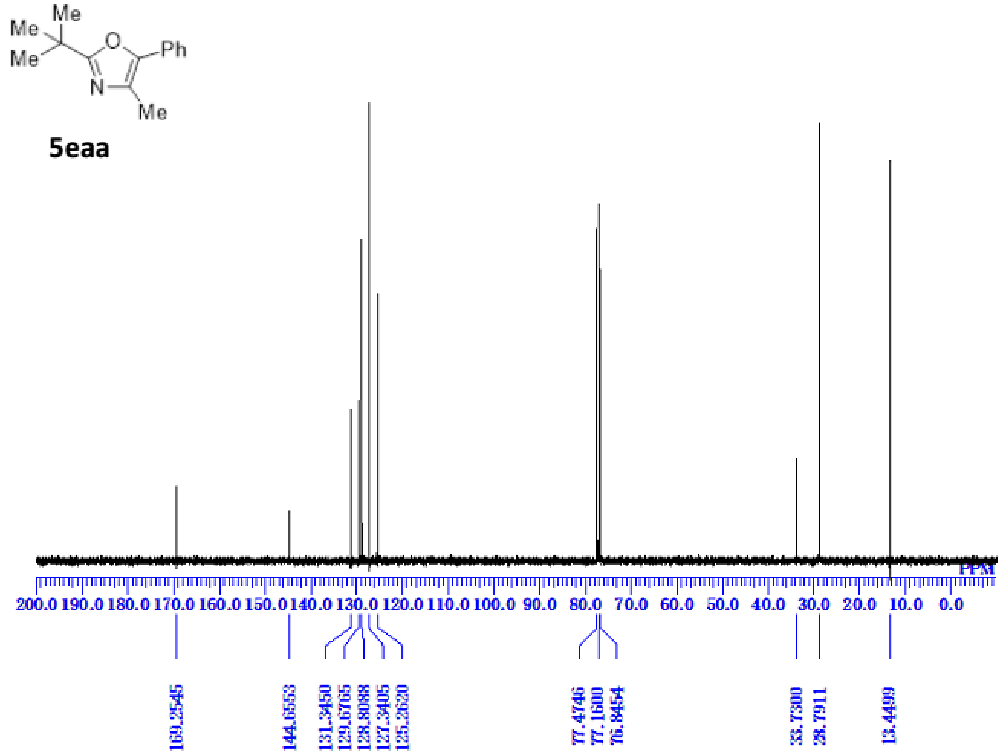

2, 2'-(1,4-Phenylene) bis(4-methyl-5-phenyl)oxazole (5faa)

^{1}H NMR (600 MHz, CDCl$_3$)

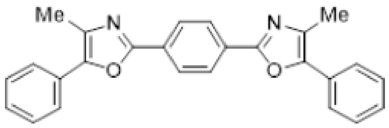

5faa

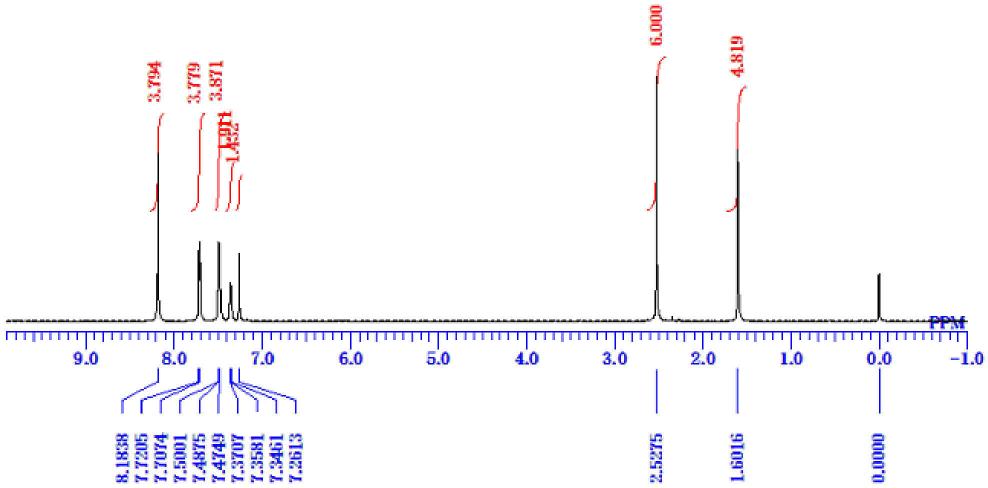

^{13}C NMR (150 MHz, CDCl$_3$)

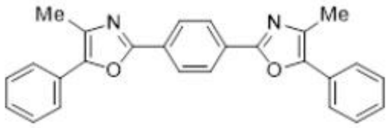

5faa

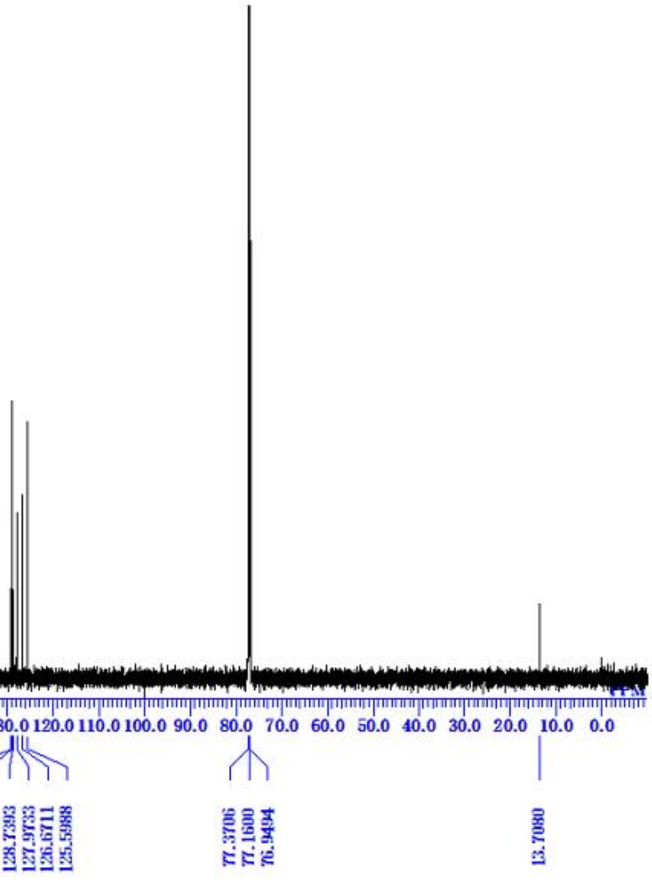

4-Benzyl-2,5-diphenyloxazole (5aba)

^{1}H NMR (600 MHz, $CDCl_3$)

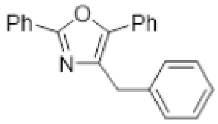

5aba

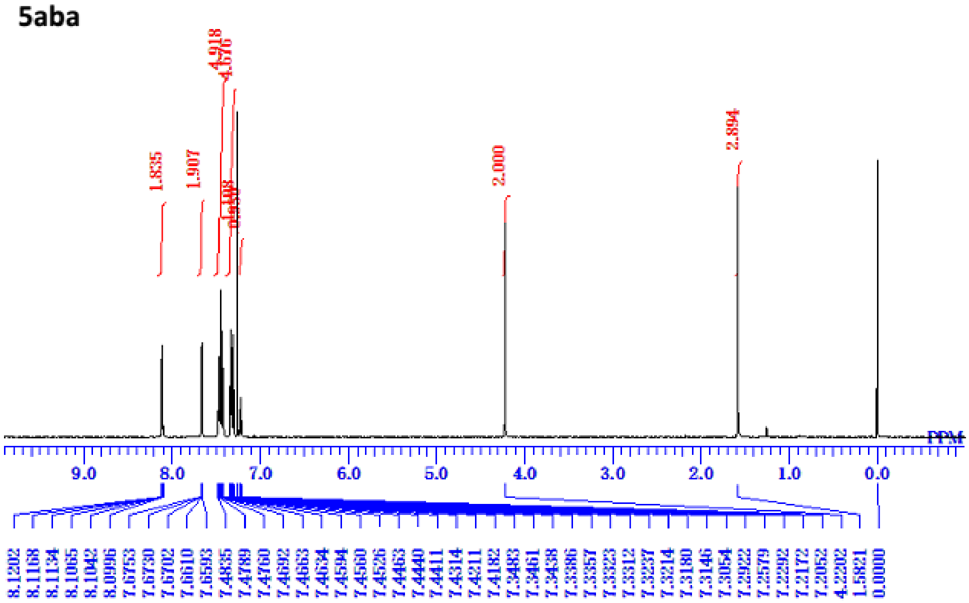

^{13}C NMR (150 MHz, $CDCl_3$)

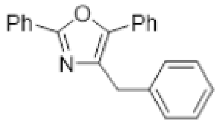

5aba

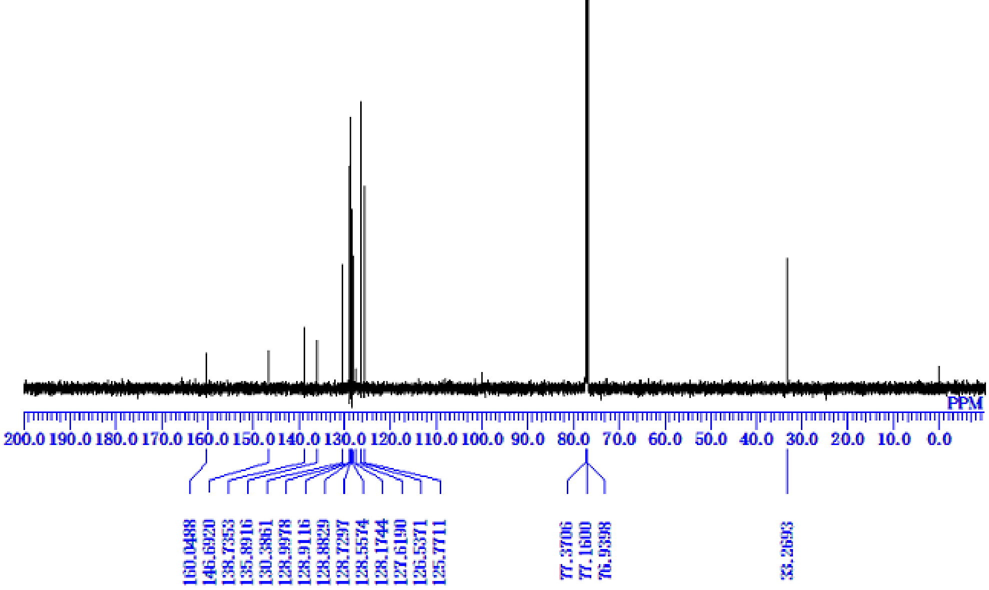

4-Isopropyl-2,5-diphenyloxazole (5aca)

^{1}H NMR (600 MHz, $CDCl_3$)

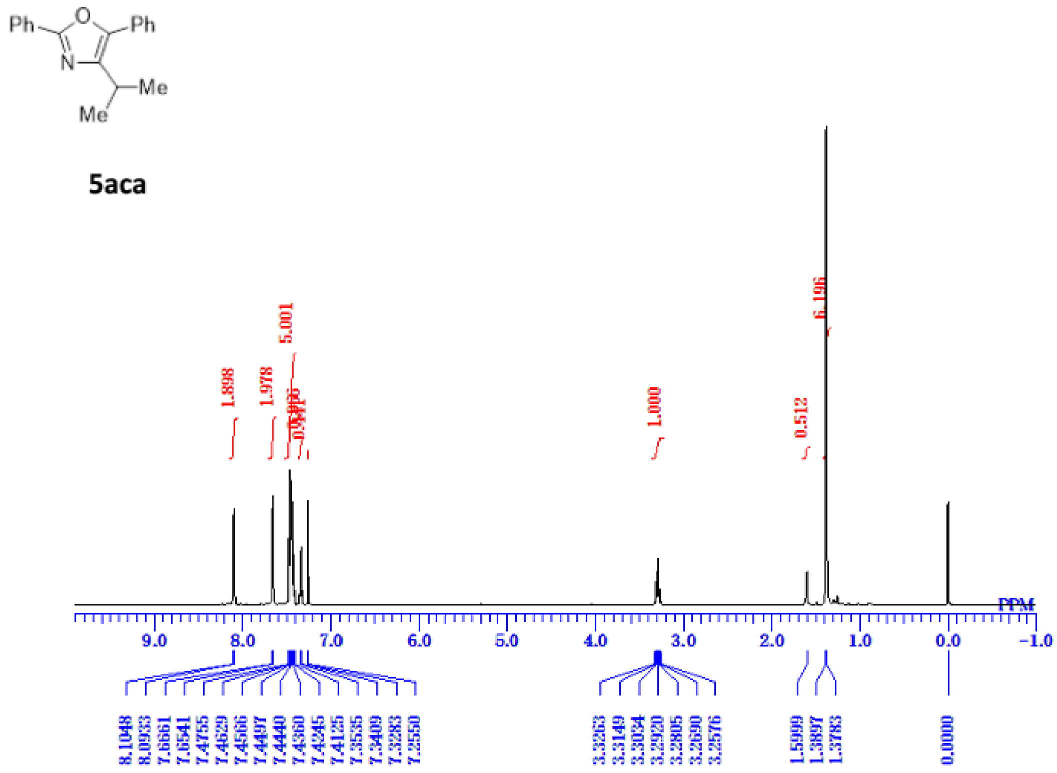

^{13}C NMR (150 MHz, $CDCl_3$)

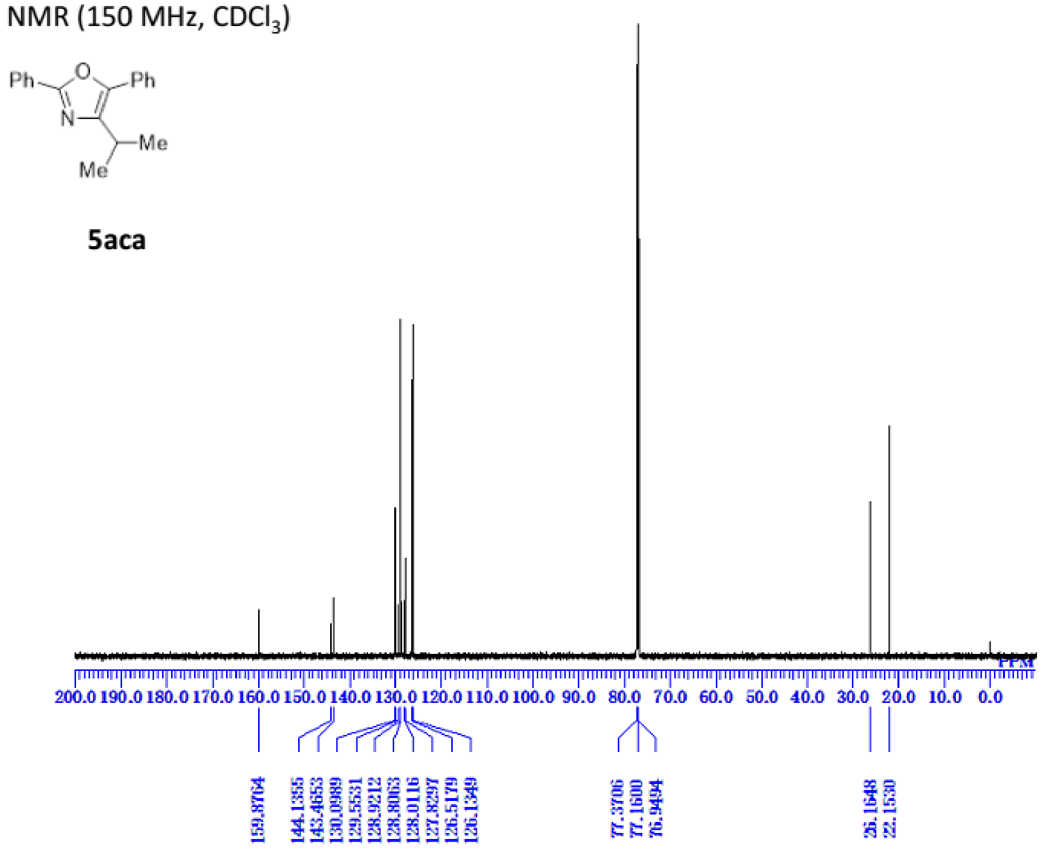

4-Isobutyl-2,5-diphenyloxazole (5ada)

^{1}H NMR (600 MHz, $CDCl_3$)

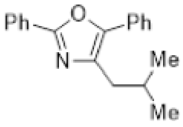

5ada

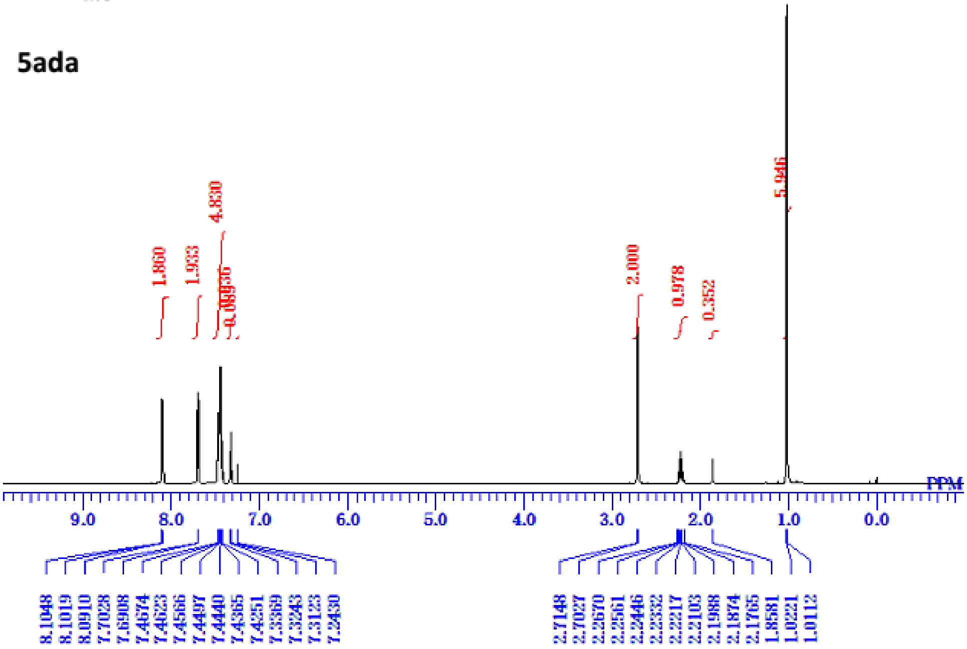

^{13}C NMR (150 MHz, $CDCl_3$)

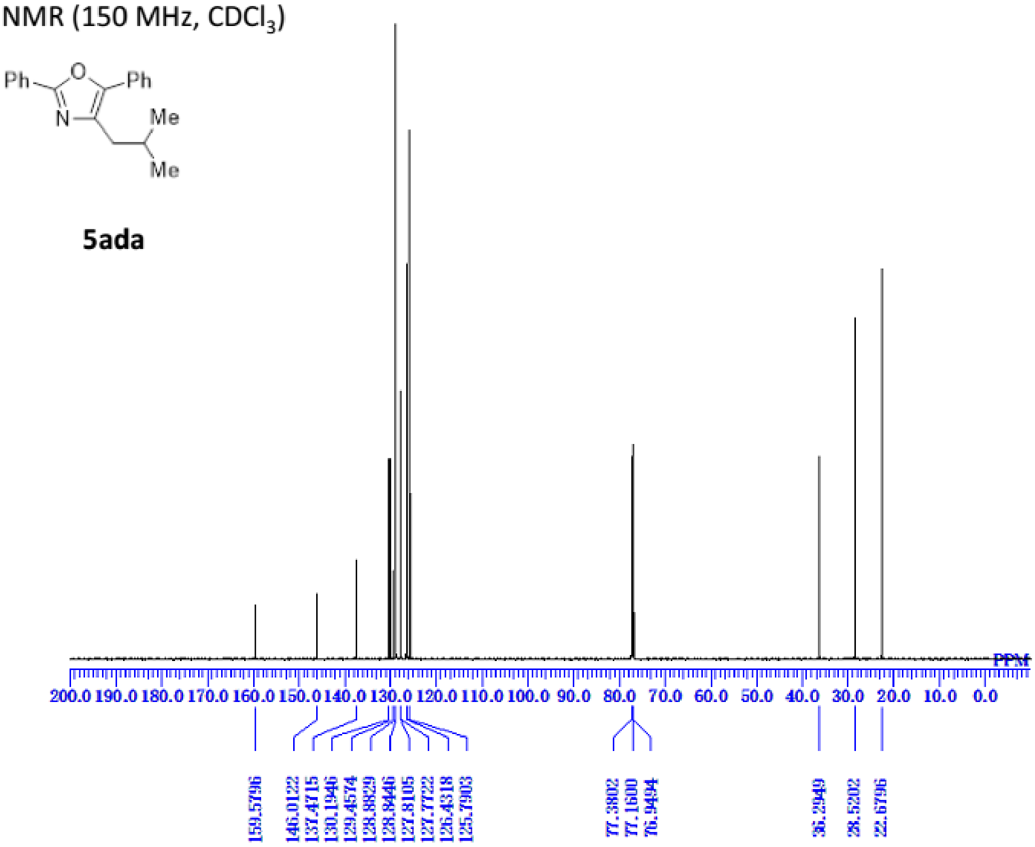

4-(2-(Methylthio)ethyl)-2,5-diphenyloxazole (5aea)

^{1}H NMR (600 MHz, $CDCl_3$)

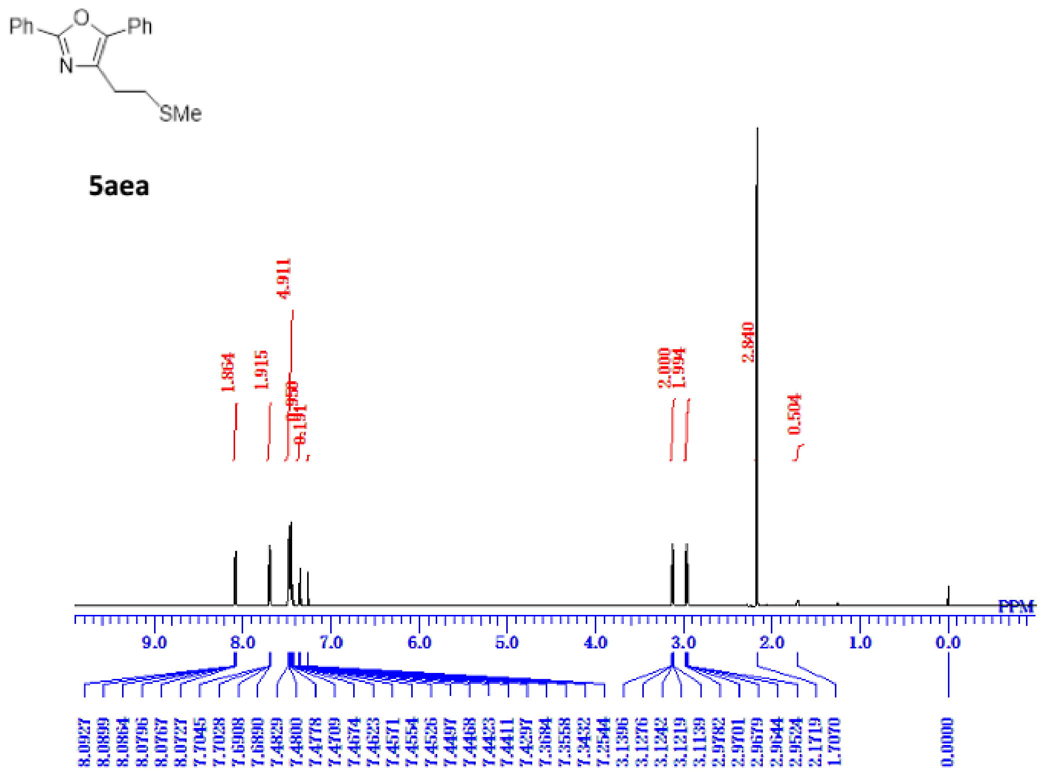

^{13}C NMR (150 MHz, $CDCl_3$)

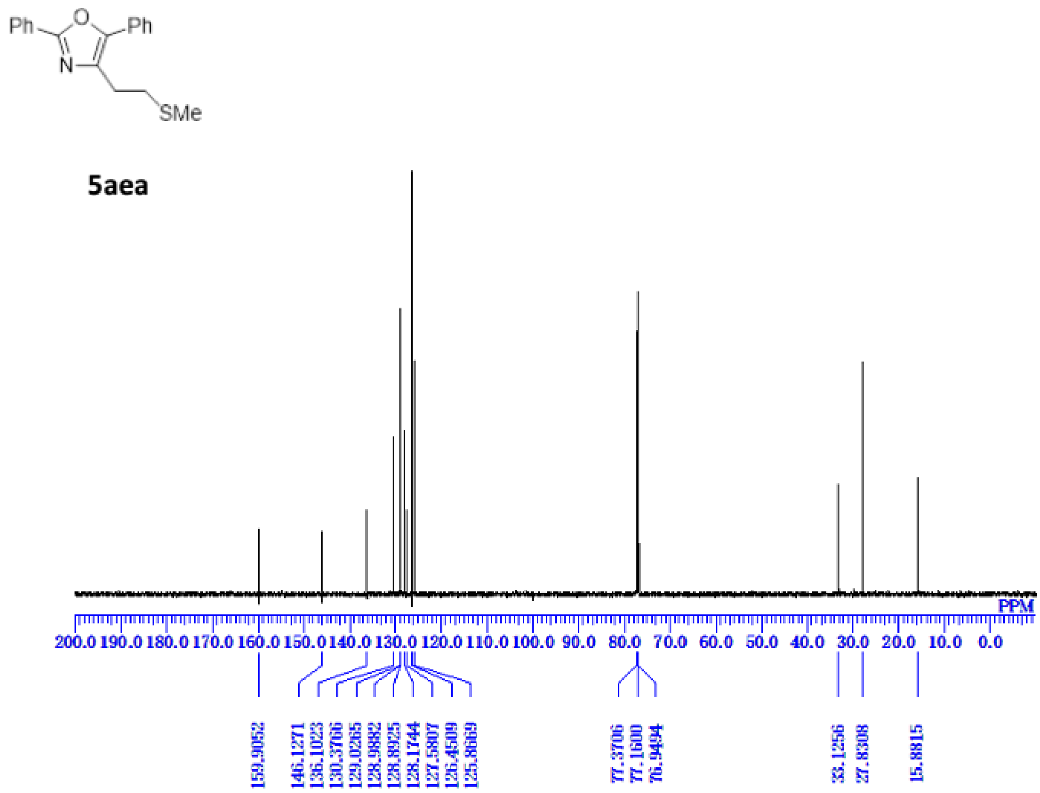

2,4,5-Triphenyloxazole (5afa)

^1H NMR (600 MHz, $CDCl_3$)

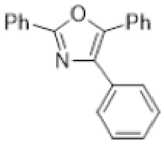

5afa

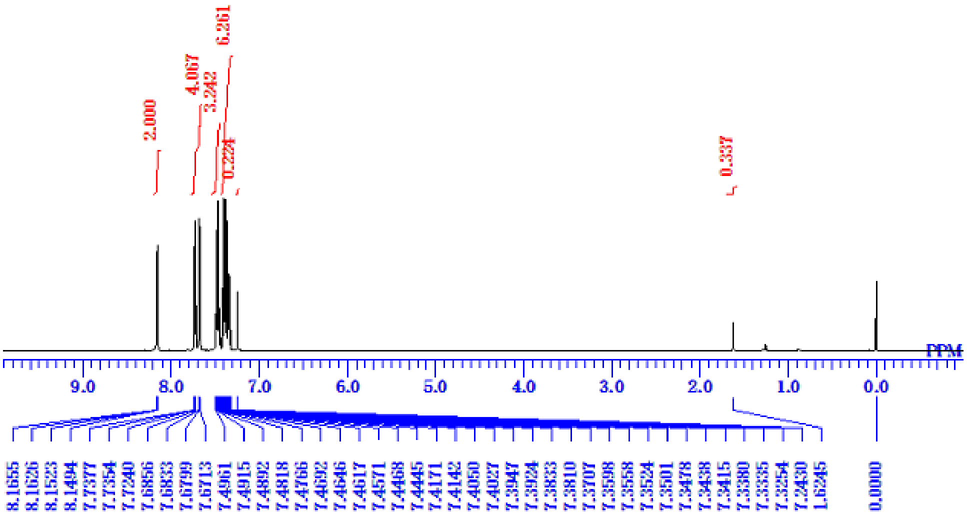

^{13}C NMR (150 MHz, $CDCl_3$)

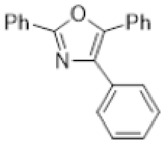

5afa

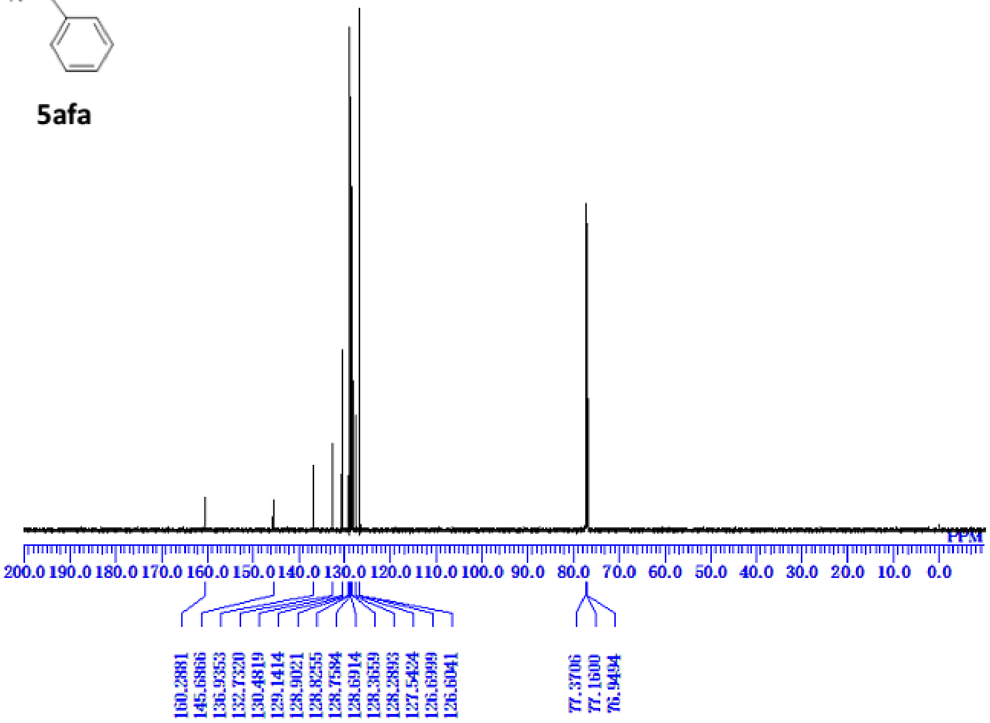

Supplement: File 1 — General information, Table S1, experimental procedure and characterization data for products, and 1H and 13C NMR spectra. [file Beilstein_J_Org_Chem-13-1478-s001.pdf]
